# Supplementary material for: Atypical Centriolar Composition Correlates with Internal Fertilization in Fish
Source: Cells. 2022 Feb 22;11(5):758. doi: 10.3390/cells11050758 (PMC8909020; doi:10.3390/cells11050758)
Supplement: Supplementary file 1 [file cells-11-00758-s001.zip › cells-1576196-supplementary.pdf]

Supplementary Table S1- List of studies fish spices included in the current analysis

Phylogeny column - coped from NCBI taxonomy
Fish Specie (Common name) - as found in the reference and NCBI taxonomy
Centriole references – The paper and books that used to interprets the centriole number and status
Centrioles number – Canonical centriole number in the sperm
PC – The PC status in the studies species
DC – The DC status in the studies species
Reproduction mode – the type of fertilization (internal or external)
Reproduction references – The paper and books that used to identify if fertilization is internal or external

Color code:
Internal fertlisation with atypical centriole
Internal fertlisation with 1 centriole
External fertlisation with 1 centrioles
Internal fertlisation with 2 centriole
External fertlisation with 2 centrioles

Phylogeny	Fish Specie	Centriole references	Centrioles number	PC	DC	Reproduction	Reproduction reference
Vertebrata; Cyclostomata; Hyperoartia; Petromyzontiformes; Petromyzontidae; Lampetra	*Lampetra planeri*<br>*Lampetra fluviatilis*<br>*Lampetra japonica*	(Jamieson, 1991)<br>(Jamieson, 1991)<br>(Jamieson, 1991)	2	Canonical	Canonical	External spawning	(Malmqvist, 1980)
Vertebrata; Cyclostomata; Myxini; Myxiniformes; Myxinidae; Eptatretinae; Eptatretus	*Eptatretus burgeri* (Inshore hagfish)	(Morisawa, 2005)	2	Canonical	Canonical DC is present	external	(Powell et al., 2005)
Vertebrata; Gnathostomata; Chondrichthyes (cartilaginous fishes); Elasmobranchii; Selachii; Squalomorphii; Squaliformes; Squalidae; Squalus	*Squalus suckleyi* (Pacific spiny dogfish)	(Stanley, 1971)	2	Canonical	Canonical (in spermatid only)	Internal - viviparous - no sperm casting	(Demirhan and Seyhan, 2006)
Vertebrata; Gnathostomata; Chondrichthyes; Elasmobranchii; Batoidea; Rhinopristiformes; Glaucostegidae; Glaucostegusz	*Rhinobatus cemiculus*	(Mattei, 1970)	2	Canonical	Canonical	Internal – Gustation- no spermcasting	(Diatta et al., 2004)
Vertebrata; Gnathostomata; Chondrichthyes (cartilaginous fishes); Holocephali; Chimaeriformes; Chimaeridae; Hydrolagus	*Hydrolagus colliei*	(Stanley, 1983)	1 (confirmed)	Undetected	Canonical	Internal - no sperm casting	(Reist, 2019)
Vertebrata; Gnathostomata; Teleostomi; Euteleostomi; Actinopterygii; Actinopteri; Chondrostei; Acipenseriformes; Acipenseroidei; Acipenseridae; Acipenserinae; Acipenserini; Acipenser	*Acipenser persicus* (Persian sturgeon) ;<br>*Acipenser transmontanus*	(Hatef et al., 2011)<br>(Jamieson, 1991)	2	Canonical	Canonical	External	(Ruban et al., 2011)
	Acipenser ruthenus (Starlet sturgeon)	(Psenicka et al., 2008)<br>(Psenicka et al., 2009)	2	Canonical	Canonical	External	(Niksirat et al., 2017)
	Acipenser baerii (Siberian sturgeon)	(Psenicka et al., 2007)<br>(Psenicka et al., 2008)	2	Canonical	Canonical	External	(Wei et al., 2007)
	Acipenser sinensis (Chinese sturgeon)	(Wei et al., 2007)	2	Canonical	Canonical	External	(Wei et al., 2007)
Vertebrata; Gnathostomata; Teleostomi; Euteleostomi; Actinopterygii; Actinopteri; Chondrostei; Acipenseriformes; Acipenseroidei; Acipenseridae; Acipenserinae; Scaphirhychini; Scaphirhynchus	*Scaphirhynchus albus* (Pallid sturgeon)	(DiLauro et al., 2001)	2	Canonical	Canonical	External-Spawning	(Flamio Jr et al., 2021)
Vertebrata; Gnathostomata; Teleostomi; Euteleostomi; Actinopterygii; Actinopteri; Chondrostei; Acipenseriformes; Acipenseroidei; Polyodontidae; Polyodontinae; Polyodontini; Polyodon	*Polyodon spathula* (Mississippi paddlefish)	(Zarnescu, 2005)	2	Canonical	Canonical	External - Spawning	(Needham, 1965)

Vertebrata; Gnathostomata; Teleostomi; Euteleostomi; Actinopterygii; Cladistia; Polypteriformes; Polypteridae; Polypterus	*Polypterus senegalus*	(Jamieson, 1991)	2	Canonical	Canonical	External spawning	(Subamia and Sugito, 2008)
Gnathostomata; Teleostomi; Euteleostomi; Actinopterygii; Actinopteri; Neopterygii; Holostei; Semionotiformes; Lepisosteidae; Lepisosteus	*Lepisosteus osseus*	(Jamieson, 1991)	2	Canonical but with modification. The PC is made up of 9 triplets but is terminated at one of its ends by 9 doublets, which we interpret as an abortive flagellum	Canonical	External spawning	(Goff, 1984)
Gnathostomata; Teleostomi; Euteleostomi; Actinopterygii; Actinopteri; Neopterygii; Holostei; Amiiformes; Amiidae; Amia	*Amia calva* (Bowfin)	(Jamieson, 1991)	2	Canonical	Canonical	External spawning	(Koch et al., 2009)
Gnathostomata; Teleostomi; Euteleostomi; Actinopterygii; Actinopteri; Neopterygii; Holostei; Semionotiformes	*Lepisosteiformes* (Gars)	(Jamieson, 1991)	2	Canonical	Canonical	External spawning	(McEachran and Fechhelm, 2021)
Gnathostomata; Teleostomi; Euteleostomi; Actinopterygii; Actinopteri; Neopterygii; Teleostei; Osteoglossocephalai; Osteoglossocephala; Osteoglossomorpha; Osteoglossiformes; Mormyridae	*Brienomyrus niger*<br>*Marcusenius senegalenis*<br>*Hyperopsius bebe*<br>*Mormyrus rume*<br>*Petrocephalus bovei*	(Jamieson, 1991)	2	Canonical	Canonical	External spawning	(Alhassan et al., 2014)
Gnathostomata; Teleostomi; Euteleostomi; Actinopterygii; Actinopteri; Neopterygii; Teleostei; Osteoglossocephalai; Osteoglossocephala; Osteoglossomorpha; Osteoglossiformes; Gymnarchidae; Gymnarchus	*Gymnarchus niloticus*	(Jamieson, 1991)	2	Canonical	Canonical	External spawning	(Little et al., 1993a)
Gnathostomata; Teleostomi; Euteleostomi; Actinopterygii; Actinopteri	*Neopterygii*	(Jamieson, 1991)	2	Canonical	Canonical	External spawning	(Xu and Zhao, 2016)
Gnathostomata; Teleostomi; Euteleostomi; Actinopterygii; Actinopteri; Neopterygii; Teleostei; Osteoglossocephalai; Clupeocephala; Otomorpha; Ostariophysi; Anotophysi; Gonorynchiformes; Chanoidei; Chanidae; Chanos	*Chanos chanos*	(Jamieson, 1991)	2	Canonical	Canonical	External spawning	(Lee et al., 1986)
Gnathostomata; Teleostomi; Euteleostomi; Actinopterygii; Actinopteri; Neopterygii; Teleostei; Osteoglossocephalai; Clupeocephala; Otomorpha; Ostariophysi; Otophysi; Cypriniphysae; Cypriniformes; Cyprinoidei; Leuciscidae; Leuciscinae	*Leuciscus victorianus*	(Jamieson, 1991)	2	Canonical	Canonical	External spawning	(Rutaisire, 2003)
Gnathostomata; Teleostomi; Euteleostomi; Actinopterygii; Actinopteri; Neopterygii; Teleostei; Osteoglossocephalai; Clupeocephala; Otomorpha; Ostariophysi; Otophysi; Cypriniphysae; Cypriniformes; Cobitoidei; Cobitidae; Cobitinae; Misgurnus	*Misgurnus anguillicaudatus*	(Jamieson, 1991)	2	Canonical	Canonical	External spawning	(Fujimoto et al., 2008)
Vertebrata; Gnathostomata; Teleostomi; Euteleostomi; Actinopterygii; Actinopteri; Neopterygii; Teleostei; Osteoglossocephalai; Clupeocephala; Otomorpha; Ostariophysi; Otophysi; Cypriniphysae; Cypriniformes; Cobitoidei; Cobitidae; Cobitinae; Misgurnus	*Misgurnus fossilis*	(Alavi et al., 2013) (Nadezhdina et al., 2001)	2	Canonical	Canonical	External spawning	(Kostomarova, 1991)
Vertebrata; Gnathostomata; Teleostomi; Euteleostomi; Actinopterygii; Actinopteri; Neopterygii; Teleostei; Osteoglossocephalai; Clupeocephala; Otomorpha; Ostariophysi; Otophysi; Cypriniphysae; Cypriniformes; Cyprinoidei; Danionidae; Danioninae; Danio	*Danio rerio* (Zebrafish)	(Rupik et al., 2011) (Zhang et al., 2014)	2	Canonical	Canonical	Externa spawning	(Meinelt et al., 1999)
Vertebrata; Gnathostomata; Teleostomi; Euteleostomi; Actinopterygii; Actinopteri; Neopterygii; Teleostei; Osteoglossocephalai; Clupeocephala; Otomorpha; Ostariophysi; Otophysi; Cypriniphysae; Cypriniformes; Cyprinoidei; Cyprinidae; Cyprininae; Cyprinus	*Cyprinus carpio* (Common carp)	(Pšenička et al., 2008)	2	Canonical	Canonical	External- Spawning	(Aliniya et al., 2013)
Vertebrata; Gnathostomata; Teleostomi; Euteleostomi; Actinopterygii; Actinopteri; Neopterygii; Teleostei; Osteoglossocephalai; Clupeocephala; Otomorpha; Ostariophysi; Otophysi; Cypriniphysae; Cypriniformes; Cobitoidei; Cobitidae; Cobitinae; Misgurnus	*Misgurnus anguillicaudatus* (Oriental weatherfish)	(Ohta et al., 1993)	2	Canonical	Canonical	Internal- Insemination	(Fujimoto et al., 2006)
Vertebrata; Gnathostomata; Teleostomi; Euteleostomi;	*Acheilognathus rhombeus*	(Ohta et al., 1993)	2	Canonical	Canonical	External- Spawning	(Kawamura and

Actinopterygii; Actinopteri; Neopterygii; Teleostei; Osteoglossocephalai; Clupeocephala; Otomorpha; Ostariophysi; Otophysi; Cypriniphysae; Cypriniformes; Cyprinoidei; Acheilognathidae; Acheilognathus							Uehara, 2005)
Vertebrata; Gnathostomata; Teleostomi; Euteleostomi; Actinopterygii; Actinopteri; Neopterygii; Teleostei; Osteoglossocephalai; Clupeocephala; Otomorpha; Ostariophysi; Otophysi; Cypriniphysae; Cypriniformes; Cyprinoidei; Tincidae; Tinca	*Tinca tinca* (Tench)	(Psenicka et al., 2006)	2	Canonical	Canonical	Internal- Insemination (Artificial)	(Linhart et al., 2006)
Vertebrata; Gnathostomata; Teleostomi; Euteleostomi; Actinopterygii; Actinopteri; Neopterygii; Teleostei; Osteoglossocephalai; Clupeocephala; Otomorpha; Ostariophysi; Otophysi; Cypriniphysae; Cypriniformes; Cyprinoidei; Cyprinidae; Cyprininae; Carassius	*Carassius auratus* (Goldfish)	(Ohta et al., 1993)	2	Canonical	Canonical	Internal- Insemination (artificial)	(Yamaha and Yamazaki, 2002)
Vertebrata; Gnathostomata; Teleostomi; Euteleostomi; Actinopterygii; Actinopteri; Neopterygii; Teleostei; Osteoglossocephalai; Clupeocephala; Otomorpha; Ostariophysi; Otophysi; Cypriniphysae; Cypriniformes; Cyprinoidei; Acheilognathidae; Rhodeus	*Rhodeus ocellatus* (Rosy bitterling)	(Ohta, 1991)	2	Canonical	Canonical	Internal - Insemination	(Ueno and Arimoto, 1982)
Vertebrata; Gnathostomata; Teleostomi; Euteleostomi; Actinopterygii; Actinopteri; Neopterygii; Teleostei; Osteoglossocephalai; Clupeocephala; Otomorpha; Ostariophysi; Otophysi; Cypriniphysae; Cypriniformes; Cyprinoidei; Xenocyprididae; Xenocypridinae; Hemiculter	*Hemiculter eigenmanni*	(Kim, 2019)	2	Canonical	Canonical	External	(Kim, 2019)
Vertebrata; Gnathostomata; Teleostomi; Euteleostomi; Actinopterygii; Actinopteri; Neopterygii; Teleostei; Osteoglossocephalai; Clupeocephala; Otomorpha; Ostariophysi; Otophysi; Cypriniphysae; Cypriniformes; Cyprinoidei; Cyprinidae; Acrossocheilinae; Acrossocheilus	*Acrossocheilus fasciatus*	(Fu et al., 2016)	2	Canonical	Canonical	External - spawning	(ZHANG and JIANG, 2010)
Vertebrata; Gnathostomata; Teleostomi; Euteleostomi; Actinopterygii; Actinopteri; Neopterygii; Teleostei; Osteoglossocephalai; Clupeocephala; Otomorpha; Ostariophysi; Otophysi; Characiphysae; Characiformes; Characoidei; Lebiasinidae; Lebiasininae	*Lebiasina*	(Santana et al., 2013)	2	Canonical	Canonical	External- spawning	(Urbano-Bonilla et al., 2016)
Vertebrata; Gnathostomata; Teleostomi; Euteleostomi; Actinopterygii; Actinopteri; Neopterygii; Teleostei; Osteoglossocephalai; Clupeocephala; Otomorpha; Ostariophysi; Otophysi; Cypriniphysae; Cypriniformes; Cyprinoidei	*Cyprinidae*	(Fürböck et al., 2009)	2	Canonical	Canonical	External- spawning	(Hontela and Stacey, 2019)
Vertebrata; Gnathostomata; Teleostomi; Euteleostomi; Actinopterygii; Actinopteri; Neopterygii; Teleostei; Osteoglossocephalai; Clupeocephala; Otomorpha; Ostariophysi; Otophysi; Cypriniphysae; Cypriniformes; Cyprinoidei; Leuciscidae; Leuciscinae; Chondrostoma	*Chondrostoma nasus*	(Fürböck et al., 2010)	2	Canonical	Canonical	External spawning	(Peňáz, 1996)
Gnathostomata; Teleostomi; Euteleostomi; Actinopterygii; Actinopteri; Neopterygii; Teleostei; Osteoglossocephalai; Clupeocephala; Otomorpha; Ostariophysi; Otophysi; Cypriniphysae; Cypriniformes; Cobitoidei; Cobitidae; Cobitinae;	*Cobitidae*	(Jamieson, 1991)	2	Canonical	Canonical	External spawning	(Bohlen, 2008)
Gnathostomata; Teleostomi; Euteleostomi; Actinopterygii; Actinopteri; Neopterygii; Teleostei; Osteoglossocephalai; Clupeocephala; Otomorpha; Ostariophysi; Otophysi; Characiphysae; Characiformes; Characoidei; Parodontidae; Apareiodon	*Apareiodon affinis*	(Jamieson, 1991)	2	Canonical	Canonical	External spawning	(Fonseca Ratton et al., 2003)
Gnathostomata; Teleostomi; Euteleostomi; Actinopterygii; Actinopteri; Neopterygii; Teleostei; Osteoglossocephalai; Clupeocephala; Otomorpha; Ostariophysi; Otophysi; Characiphysae; Characiformes; Characoidei; Curimatidae	*Psectrogaster rutiloides* (Chiochio)	(Jamieson, 1991)	2	Canonical	Canonical	External spawning	(Riofrio-Quijandría et al., 2017)
Gnathostomata; Teleostomi; Euteleostomi; Actinopterygii; Actinopteri; Neopterygii; Teleostei; Osteoglossocephalai; Clupeocephala; Otomorpha; Ostariophysi; Otophysi; Characiphysae; Characiformes; Characoidei; Prochilodontidae	*Pampus argenteus*	(Jamieson, 1991)	2	Canonical	Canonical	External spawning	{Breder, 1966 #388} By Fishbase

Gnathostomata; Teleostomi; Euteleostomi; Actinopterygii; Actinopteri; Neopterygii; Teleostei; Osteoglossocephalai; Clupeocephala; Otomorpha; Ostariophysi; Otophysi; Characiphysae; Characiformes; Characoidei; Crenuchidae; Characidiinae; Characidium	*Characidium gomesi*	(Jamieson, 1991)	2	Canonical	Canonical	Internal impregnation	(Pucci et al., 2014)
Gnathostomata; Teleostomi; Euteleostomi; Actinopterygii; Actinopteri; Neopterygii; Teleostei; Osteoglossocephalai; Clupeocephala; Otomorpha; Ostariophysi; Otophysi; Characiphysae; Characiformes; Characoidei; Hemiodontidae; Anodus	*Anodus elongatus*	(Jamieson, 1991)	2	Canonical	Canonical	External spawning	(De Lima and Araujo-Lima, 2004)
Gnathostomata; Teleostomi; Euteleostomi; Actinopterygii; Actinopteri; Neopterygii; Teleostei; Osteoglossocephalai; Clupeocephala; Otomorpha; Ostariophysi; Otophysi; Characiphysae; Characiformes; Characoidei; Triportheidae; Triportheus	*triportheus paranensis*	(Jamieson, 1991)	2	Canonical	Canonical	External spawning	(Mariac et al., 2021) By *Triportheus albus*
Gnathostomata; Teleostomi; Euteleostomi; Actinopterygii; Actinopteri; Neopterygii; Teleostei; Osteoglossocephalai; Clupeocephala; Otomorpha; Ostariophysi; Otophysi; Characiphysae; Characiformes; Characoidei; Serrasalmidae; Piaractus	*Piaractus mesopotamicus*	(Jamieson, 1991)	2	Canonical	Canonical	External spawning	(Romagosa et al., 1990)
Gnathostomata; Teleostomi; Euteleostomi; Actinopterygii; Actinopteri; Neopterygii; Teleostei; Osteoglossocephalai; Clupeocephala; Otomorpha; Ostariophysi; Otophysi; Characiphysae; Characiformes; Characoidei; Characidae; Aphyocharacinae; Aphyocharax	*Aphyocharax anisitsi*	(Jamieson, 1991)	2	Canonical	Canonical	external	(Gonçalves et al., 2005)
Gnathostomata; Teleostomi; Euteleostomi; Actinopterygii; Actinopteri; Neopterygii; Teleostei; Osteoglossocephalai; Clupeocephala; Otomorpha; Ostariophysi; Otophysi; Characiphysae; Characiformes; Characoidei; Characidae; Cheirodontinae; Serrapinnus	*Serrapinnus kriegi*	(Jamieson, 1991)	2	Canonical	Canonical	External spawning	(Felicio et al., 2021)
Gnathostomata; Teleostomi; Euteleostomi; Actinopterygii; Actinopteri; Neopterygii; Teleostei; Osteoglossocephalai; Clupeocephala; Otomorpha; Ostariophysi; Otophysi; Characiphysae; Characiformes; Characoidei; Characidae;	*Mimagoniates microlepis*<br>*Mimagoniates barberi*<br>*Scopaeocharax rhinodus*	(Jamieson, 1991)	2	Canonical	Canonical	Internal inseminating	(Azevedo et al., 2016)
Gnathostomata; Teleostomi; Euteleostomi; Actinopterygii; Actinopteri; Neopterygii; Teleostei; Osteoglossocephalai; Clupeocephala; Otomorpha; Ostariophysi; Otophysi; Characiphysae; Characiformes; Characoidei; Characidae; Glandulocaudinae; Diapoma	*Diapoma speculiferum*	(Jamieson, 1991)	2	Canonical	Canonical	Internal inseminating	(Azevedo et al., 2000)
Gnathostomata; Teleostomi; Euteleostomi; Actinopterygii; Actinopteri; Neopterygii; Teleostei; Osteoglossocephalai; Clupeocephala; Otomorpha; Ostariophysi; Otophysi; Characiphysae; Characiformes; Characoidei; Characidae; Glandulocaudinae; Corynopoma	*Corynopoma riisei*	(Jamieson, 1991)	2	Canonical	Canonical	Internal inseminating	(Pecio et al., 2007)
Gnathostomata; Teleostomi; Euteleostomi; Actinopterygii; Actinopteri; Neopterygii; Teleostei; Osteoglossocephalai; Clupeocephala; Otomorpha; Ostariophysi; Otophysi; Characiphysae; Characiformes; Characoidei; Characidae; Glandulocaudinae; Pseudocorynopoma	*Pseudocorynopomadoriae*	(Jamieson, 1991)	2	Canonical	Canonical	Internal inseminating	(Azevedo et al., 2000)
Gnathostomata; Teleostomi; Euteleostomi; Actinopterygii; Actinopteri; Neopterygii; Teleostei; Osteoglossocephalai; Clupeocephala; Otomorpha; Ostariophysi; Otophysi; Characiphysae; Characiformes; Characoidei; Characidae; Stevardiinae	*Chrysobrycon*	(Jamieson, 1991)	2	Canonical	Canonical	Internal inseminating	(Baicere-Silva et al., 2011)
Gnathostomata; Teleostomi; Euteleostomi; Actinopterygii; Actinopteri; Neopterygii; Teleostei; Osteoglossocephalai; Clupeocephala; Otomorpha; Ostariophysi; Otophysi; Characiphysae; Characiformes; Characoidei; Characidae; Cheirodontinae; Odontostilbe	*Odontostilbe dialeptura*	(Jamieson, 1991)	2	Canonical	Canonical	External	(Oliveira et al., 2010) By *Odontostilbe pequira*
Gnathostomata; Teleostomi; Euteleostomi; Actinopterygii; Actinopteri; Neopterygii; Teleostei; Osteoglossocephalai; Clupeocephala; Otomorpha; Ostariophysi; Otophysi; Characiphysae; Characiformes; Characoidei; Characidae; Characidae incertae sedis; Brittanichthys	*Brittanichthys axelrodi*	(Jamieson, 1991)	2	Canonical	Canonical	Internal inseminating	(Javonillo et al., 2007)

Gnathostomata; Teleostomi; Euteleostomi; Actinopterygii; Actinopteri; Neopterygii; Teleostei; Osteoglossocephalai; Clupeocephala; Otomorpha; Ostariophysi; Otophysi; Characiphysae; Characiformes; Characoidei; Characidae; Stevardiinae; Bryconadenos	*Bryconadenos tanaothoros*	(Jamieson, 1991)	2	Canonical	Canonical	Internal insemination	(Weitzman et al., 2005)
Gnathostomata; Teleostomi; Euteleostomi; Actinopterygii; Actinopteri; Neopterygii; Teleostei; Osteoglossocephalai; Clupeocephala; Otomorpha; Ostariophysi; Otophysi; Characiphysae; Siluriformes; Diplomystoidei; Diplomystidae; Diplomystes	*Diplomystes mesembrinus*	(Jamieson, 1991)	2	Canonical	Canonical	external	(Quagio-Grassiotto et al., 2001)
Gnathostomata; Teleostomi; Euteleostomi; Actinopterygii; Actinopteri; Neopterygii; Teleostei; Osteoglossocephalai; Clupeocephala; Otomorpha; Ostariophysi; Otophysi; Characiphysae; Siluriformes; Siluroidei; Cetopsidae; Cetopsis	*Cetopsis coecutiens*	(Jamieson, 1991)	2	Canonical	Canonical	external	(Smith et al., 2013) By *Cetopsis gobioides*
Gnathostomata; Teleostomi; Euteleostomi; Actinopterygii; Actinopteri; Neopterygii; Teleostei; Osteoglossocephalai; Clupeocephala; Otomorpha; Ostariophysi; Otophysi; Characiphysae; Siluriformes; Loricarioidei; Trichomycteridae	*Trichomycterus reinhardti*<br>*Trichomycterus sp.*	(Jamieson, 1991)	2	Canonical	Canonical	External spawning	(Marraro et al., 2005) By *Trichomycterus corduvense*
Gnathostomata; Teleostomi; Euteleostomi; Actinopterygii; Actinopteri; Neopterygii; Teleostei; Osteoglossocephalai; Clupeocephala; Otomorpha; Ostariophysi; Otophysi; Characiphysae; Siluriformes; Loricarioidei; Nematogenyidae; Nematogenys	*Nematogenys inermis*	(Jamieson, 1991)	2	Canonical	Canonical	External spawning	(Vila and Habit, 2015)
Gnathostomata; Teleostomi; Euteleostomi; Actinopterygii; Actinopteri; Neopterygii; Teleostei; Osteoglossocephalai; Clupeocephala; Otomorpha; Ostariophysi; Otophysi; Characiphysae; Siluriformes; Loricarioidei; Callichthyidae;	*Aspidoras poecilus*<br>*Corydoras aeneus*<br>*Corydoras flaveolus*	(Jamieson, 1991)	2	Canonical	Canonical	External spawning	(Araujo and Garutti, 2002)
Gnathostomata; Teleostomi; Euteleostomi; Actinopterygii; Actinopteri; Neopterygii; Teleostei; Osteoglossocephalai; Clupeocephala; Otomorpha; Ostariophysi; Otophysi; Characiphysae; Siluriformes; Loricarioidei	*Ancistrus triradiatus*<br>*Hypostomus ancistroides*<br>*Loricariichthys platymetopon*	(Jamieson, 1991)	2	Canonical	Canonical	External spawning	(Collazos-Lasso and Arias-Castellanos, 2009)
Gnathostomata; Teleostomi; Euteleostomi; Actinopterygii; Actinopteri; Neopterygii; Teleostei; Osteoglossocephalai; Clupeocephala; Otomorpha; Ostariophysi; Otophysi; Characiphysae; Siluriformes; Siluroidei	*Amblycipitidae*<br>*Pseudopimelodidae*	(Jamieson, 1991)	2	Canonical	Canonical	External spawning	(Santos et al., 2013)
Gnathostomata; Teleostomi; Euteleostomi; Actinopterygii; Actinopteri; Neopterygii; Teleostei; Osteoglossocephalai; Clupeocephala; Otomorpha; Ostariophysi; Otophysi; Characiphysae; Siluriformes; Siluroidei	*Ictaluridae*	(Jamieson, 1991)	2	Canonical	Canonical	External spawning	(Sneed and Clemens, 1963)
Gnathostomata; Teleostomi; Euteleostomi; Actinopterygii; Actinopteri; Neopterygii; Teleostei; Osteoglossocephalai; Clupeocephala; Otomorpha; Ostariophysi; Otophysi; Characiphysae; Siluriformes; Siluroidei	*Siluridae*	(Jamieson, 1991)	2	Canonical	Canonical	External spawning	(Barcellos et al., 2001)
Gnathostomata; Teleostomi; Euteleostomi; Actinopterygii; Actinopteri; Neopterygii; Teleostei; Osteoglossocephalai; Clupeocephala; Otomorpha; Ostariophysi; Otophysi; Characiphysae; Siluriformes; Siluroidei; Malapteruridae	*Malapterurus*	(Jamieson, 1991)	2	Canonical	Canonical	External spawning	(A. Sabry . and et al., 2021)
Gnathostomata; Teleostomi; Euteleostomi; Actinopterygii; Actinopteri; Neopterygii; Teleostei; Osteoglossocephalai; Clupeocephala; Otomorpha; Ostariophysi; Otophysi; Characiphysae; Siluriformes; Siluroidei; Plotosidae; Plotosus	*Plotosus lineatus*	(Jamieson, 1991)	2	Canonical	Canonical	External spawning	(Matsumura et al., 2004)
Gnathostomata; Teleostomi; Euteleostomi; Actinopterygii; Actinopteri; Neopterygii; Teleostei; Osteoglossocephalai; Clupeocephala; Otomorpha; Ostariophysi; Otophysi; Characiphysae; Siluriformes; Siluroidei; Clariidae; Clarias	*Clarias anguillaris*<br>*Clarias gariepinus*<br>*Clarias lazera*	(Jamieson, 1991)	2	Canonical	Canonical	External spawning	(Idahor, 2014)

Gnathostomata; Teleostomi; Euteleostomi; Actinopterygii; Actinopteri; Neopterygii; Teleostei; Osteoglossocephalai; Clupeocephala; Otomorpha; Ostariophysi; Otophysi; Characiphysae; Siluriformes; Siluroidei	*Schilbidae*<br>*Bagridae pimelodidae*	(Jamieson, 1991)	2	Canonical	Canonical	External spawning	(Khatun et al., 2019)
Gnathostomata; Teleostomi; Euteleostomi; Actinopterygii; Actinopteri; Neopterygii; Teleostei; Osteoglossocephalai; Clupeocephala; Otomorpha; Ostariophysi; Otophysi; Characiphysae; Gymnotiformes; Gymnotoidei; Gymnotidae; Gymnotus	*Gymnotus carapo*	(Jamieson, 1991)	2	Canonical	Canonical	External spawning	(Kirschbaum and Schugardt, 2002)
Gnathostomata; Teleostomi; Euteleostomi; Actinopterygii; Actinopteri; Neopterygii; Teleostei; Osteoglossocephalai; Clupeocephala; Otomorpha; Ostariophysi; Otophysi; Characiphysae; Gymnotiformes; Sternopygoidei; Rhamphichthyidae; Rhamphichthys	*Rhamphichthys hahni.*	(Jamieson, 1991)	2	Canonical	Canonical	External spawning	(Kirschbaum and Schugardt, 2002) By *Rhamphichthys sp.*
Gnathostomata; Teleostomi; Euteleostomi; Actinopterygii; Actinopteri; Neopterygii; Teleostei; Osteoglossocephalai; Clupeocephala; Otomorpha; Ostariophysi; Otophysi; Characiphysae; Siluriformes; Siluroidei; Ictaluridae	*Ictalurus*	(Jamieson, 1991)	2	Canonical	Canonical	External spawning	(Clemens and Sneed, 1957)
Gnathostomata; Teleostomi; Euteleostomi; Actinopterygii; Actinopteri; Neopterygii; Teleostei; Osteoglossocephalai; Clupeocephala; Otomorpha; Ostariophysi; Otophysi; Characiphysae; Gymnotiformes; Sternopygoidei; Apteronotidae	*Apteronotus albifrons*	(Jamieson, 1991)	2	Canonical	Canonical	External spawning	(Maulana et al., 2021)
Vertebrata; Actinopterygii; Teleostei; Otomorpha Ostariophysi; Otophysi; Characiphysae; Characiformes; Characoidei; Characidae; Cheirodontinae; Macropsobrycon	*Macropsobrycon uruguayanae*	(de Oliveira et al., 2008)	2	Canonical	Canonical	Internal-insemination	(Azevedo et al., 2010)
Vertebrata; Gnathostomata; Teleostomi; Euteleostomi; Actinopterygii; Actinopteri; Neopterygii; Teleostei; Osteoglossocephalai; Clupeocephala; Otomorpha; Ostariophysi; Otophysi; Characiphysae; Siluriformes; Siluroidei; Siluridae; Silurus	*Silurus microdorsalis* (Korean endemic slender catfish)	(Lee and Kim, 2001)	2	Canonical	Canonical	External- spawning	(Ki and Lee, 2018)
Vertebrata; Gnathostomata; Teleostomi; Euteleostomi; Actinopterygii; Actinopteri; Neopterygii; Teleostei; Osteoglossocephalai; Clupeocephala; Otomorpha; Ostariophysi; Otophysi; Characiphysae; Characiformes; Characoidei; Characidae; Characidae incertae sedis; Brittanichthys	*Brittanichthys axelrodi* (Characid fish)	(Javonillo et al., 2007)	2	Canonical	Canonical	Infternal-insemination	(Javonillo et al., 2007)
Vertebrata; Gnathostomata; Teleostomi; Euteleostomi; Actinopterygii; Actinopteri; Neopterygii; Teleostei; Osteoglossocephalai; Clupeocephala; Otomorpha; Ostariophysi; Otophysi; Characiphysae; Characiformes; Characoidei; Chilodontidae; Chilodus	*Chilodus punctatus* (Spotted headstander)	(Pecio, 2003)	2	Canonical	Canonical	External- Spawning	(Chondoma, 1979)
Vertebrata; Gnathostomata; Teleostomi; Euteleostomi; Actinopterygii; Actinopteri; Neopterygii; Teleostei; Osteoglossocephalai; Clupeocephala; Otomorpha; Ostariophysi; Otophysi; Characiphysae; Characiformes; Characoidei; Alestidae; Phenacogrammus	*Phenacogrammus interruptus* (Congo tetra)	(Pecio, 2009)	2	Canonical	Canonical	external	(Pecio, 2008)
Vertebrata; Gnathostomata; Teleostomi; Euteleostomi; Actinopterygii; Actinopteri; Neopterygii; Teleostei; Osteoglossocephalai; Clupeocephala; Otomorpha; Ostariophysi; Otophysi; Characiphysae; Characiformes; Characoidei; Characidae; Stevardiinae; Tyttocharax	*Tyttocharax tambopatensis*	(Pecio et al., 2005b)	2	Canonical	Canonical	Internal-insemination	(Pecio et al., 2005a)
Vertebrata; Gnathostomata; Teleostomi; Euteleostomi; Actinopterygii; Actinopteri; Neopterygii; Teleostei; Osteoglossocephalai; Clupeocephala; Otomorpha; Ostariophysi; Otophysi; Characiphysae; Siluriformes; Siluroidei; Auchenipteridae; Auchenipterinae;	*Trachelyopterus galeatus* (Common driftwood catfish)	(Parreira et al., 2009a)	2	Canonical	Canonical	Internal-Insemination	(Parreira et al., 2009b)

Vertebrata; Gnathostomata; Teleostomi; Euteleostomi; Actinopterygii; Actinopteri; Neopterygii; Teleostei; Osteoglossocephalai; Clupeocephala; Otomorpha; Ostariophysi; Otophysi; Characiphysae; Characiformes; Characoidei; Characidae; Characidae incertae sedis; Brittanichthys	*Brittanichthys axelrodi*	(Javonillo et al., 2007)	2	Canonical	Canonical	Internal – Insemination	(Javonillo et al., 2007)
Vertebrata; Gnathostomata; Teleostomi; Euteleostomi; Actinopterygii; Actinopteri; Neopterygii; Teleostei; Osteoglossocephalai; Clupeocephala; Otomorpha; Ostariophysi; Otophysi; Characiphysae; Characiformes; Characoidei; Characidae; Tetragonopterinae; Hemibrycon	*Hemibrycon surinamensis*	(Baicere-Silva et al., 2011)	2	Canonical	Canonical	External	(Santana et al., 2013)
Vertebrata; Gnathostomata; Teleostomi; Euteleostomi; Actinopterygii; Actinopteri; Neopterygii; Teleostei; Osteoglossocephalai; Clupeocephala; Otomorpha; Ostariophysi; Otophysi; Characiphysae; Siluriformes; Siluroidei; Pimelodidae; Pimelodus	*Pimelodus maculatus*	(Quagio-Grassiotto and Oliveira, 2008)	2	Canonica	Canonical	External- spawning induction	(Buzollo et al., 2011)
Vertebrata; Gnathostomata; Teleostomi; Euteleostomi; Actinopterygii; Actinopteri; Neopterygii; Teleostei; Osteoglossocephalai; Clupeocephala; Otomorpha; Ostariophysi; Otophysi; Characiphysae; Gymnotiformes; Sternopygoidei; Sternopygidae	Eigenmannia (gymnotiformes)	(Giora and Burns, 2011)	2	Canonical	Canonical	External- spawning	(Vischer, 1989)
Vertebrata; Gnathostomata; Teleostomi; Euteleostomi; Actinopterygii; Actinopteri; Neopterygii; Teleostei; Osteoglossocephalai; Clupeocephala; Otomorpha; Ostariophysi; Otophysi; Characiphysae; Characiformes; Characoidei; Bryconidae; Bryconinae; Brycon	*Brycon vermelha*	(Faustino et al., 2015b)	2	Canonical	Canonical	external	(Faustino et al., 2015a)
Vertebrata; Gnathostomata; Teleostomi; Euteleostomi; Actinopterygii; Actinopteri; Neopterygii; Teleostei; Osteoglossocephalai; Clupeocephala; Otomorpha; Ostariophysi; Otophysi; Characiphysae; Gymnotiformes; Gymnotoidei; Gymnotidae; Gymnotus	*Gymnotus cf. anguillaris,*	(França et al., 2007)	2	Canonical	Canonical	External	(Shahin, 2006)
Vertebrata; Gnathostomata; Teleostomi; Euteleostomi; Actinopterygii; Actinopteri; Neopterygii; Teleostei; Osteoglossocephalai; Clupeocephala; Otomorpha; Ostariophysi; Otophysi; Characiphysae; Gymnotiformes; Sternopygoidei; Hypopomidae; Brachyhypopomus	*Brachyhypopomus pinnicaudatus*	(França et al., 2007)	2	Canonical	Canonical	external	(Miranda et al., 2008)
Vertebrata; Gnathostomata; Teleostomi; Euteleostomi; Actinopterygii; Actinopteri; Neopterygii; Teleostei; Osteoglossocephalai; Clupeocephala; Otomorpha; Ostariophysi; Otophysi; Characiphysae; Siluriformes; Siluroidei; Doradidae; Acanthodoras	*Acanthodoras cataphractus*	(Quagio-Grassiotto et al., 2011a)	2	Canonical	Canonical	external	(Quagio-Grassiotto et al., 2011b)
Vertebrata; Gnathostomata; Teleostomi; Euteleostomi; Actinopterygii; Actinopteri; Neopterygii; Teleostei; Osteoglossocephalai; Clupeocephala; Otomorpha; Ostariophysi; Otophysi; Characiphysae; Characiformes; Characoidei; Alestidae; Alestes	*Alestes dentex*	(Shahin, 2006)	2	Canonical	Canonical	External	(Shahin, 2006)
Vertebrata; Gnathostomata; Teleostomi; Euteleostomi; Actinopterygii; Actinopteri; Neopterygii; Teleostei; Osteoglossocephalai; Clupeocephala; Otomorpha; Ostariophysi; Otophysi; Characiphysae; Siluriformes; Loricarioidei	*Loricariidae*	(Spadella et al., 2012)	2	Canonical	Canonical	external- spawning	(Duarte et al., 2007)
Vertebrata; Gnathostomata; Teleostomi; Euteleostomi; Actinopterygii; Actinopteri; Neopterygii; Teleostei; Osteoglossocephalai; Clupeocephala; Otomorpha; Ostariophysi; Otophysi; Characiphysae; Characiformes; Characoidei; Characidae; Stevardiinae	*Monotocheirodon*	(Menezes et al., 2013)	2	Canonical	Canonical	Internal- insemination	(Burns and Weitzman, 2006)
Vertebrata; Gnathostomata; Teleostomi; Euteleostomi; Actinopterygii; Actinopteri; Neopterygii; Teleostei; Osteoglossocephalai; Clupeocephala; Otomorpha; Ostariophysi; Otophysi; Characiphysae; Characiformes; Characoidei; Characidae; Pristellinae; Hemigrammus	*Hemigrammus erythrozonus*	(Pecio et al., 2007)	2	Canonical	Canonical	external	(Pecio et al., 2007)
Vertebrata; Gnathostomata; Teleostomi; Euteleostomi; Actinopterygii; Actinopteri; Neopterygii; Teleostei; Osteoglossocephalai; Clupeocephala; Otomorpha; Ostariophysi; Otophysi; Characiphysae; Gymnotiformes;	*Gymnotus carapo*	(Kudo et al., 1994)	2	Canonical	Canonical	External- Spawning	(Meneguelli De Souza et al., 2015)

Gymnotoidei; Gymnotidae; Gymnotus								
Gnathostomata;Teleostomi; Euteleostomi; Actinopterygii; Actinopteri; Neopterygii; Teleostei; Osteoglossocephalai; Clupeocephala; Otomorpha; Ostariophysi; Otophysi; Characiphysae; Characiformes; Characoidei; Characidae; Cheirodontinae; Paracheirodon	*Paracheirodon innesi* (Neon tetra)	(Jamieson, 1991)	2	Canonical	Canonical	External spawning	(Chapman et al., 1998)
Gnathostomata; Teleostomi; Euteleostomi; Actinopterygii; Actinopteri; Neopterygii; Teleostei; Osteoglossocephalai; Clupeocephala; Otomorpha; Ostariophysi; Otophysi; Characiphysae; Gymnotiformes; Sternopygoidei; Sternopygidae; Eigenmannia	*Eigenmannia (gymnotiformes)*	(Jamieson, 1991)	2	Canonical	Canonical	External spawning	(Kirschbaum and Schugardt, 2002) By *Eigenmannia lineata*
Gnathostomata; Teleostomi; Euteleostomi; Actinopterygii; Actinopteri; Neopterygii; Teleostei; Osteoglossocephalai; Clupeocephala; Euteleosteomorpha; Protacanthopterygii; Esociformes; Esocidae; Esox	*Esox masquinongy*	(Jamieson, 1991)	2	Canonical	Canonical	External spawning	(Diana et al., 2015)
Gnathostomata; Teleostomi; Euteleostomi; Actinopterygii; Actinopteri; Neopterygii; Teleostei; Osteoglossocephalai; Clupeocephala; Euteleosteomorpha; Protacanthopterygii	*Salmoniformes*	(Jamieson, 1991)	2	Canonical	Canonical	External spawning	(Schulz et al., 2006)
Vertebrata; Gnathostomata; Teleostomi; Euteleostomi; Actinopterygii; Actinopteri; Neopterygii; Teleostei; Osteoglossocephalai; Clupeocephala; Euteleosteomorpha; Protacanthopterygii; Galaxiiformes; Galaxiidae; Galaxiinae; Galaxiini; Galaxias	*Galaxias olidus*	(Jamieson, 1991)	No centrioles<br><br>Degraded Specimen<br><br>Not part of statistics	Undetected	Undetected	External spawning	(olidus is widespread throughout Victoria)
Gnathostomata; Teleostomi; Euteleostomi; Actinopterygii; Actinopteri; Neopterygii; Teleostei; Osteoglossocephalai; Clupeocephala; Euteleosteomorpha; Protacanthopterygii; Galaxiiformes; Galaxiidae; Galaxiinae; Galaxiini; Galaxias	*Galaxius olidus*	(Jamieson, 1991)	2	Undetected	Undetected	External spawning	(O'Connor and Koehn, 1991)
Vertebrata; Gnathostomata; Teleostomi; Euteleostomi; Actinopterygii; Actinopteri; Neopterygii; Teleostei; Osteoglossocephalai; Clupeocephala; Euteleosteomorpha; Protacanthopterygii; Salmoniformes; Salmonidae; Salmoninae; Oncorhynchus; Oncorhynchus masou	*Oncorhynchus masou formosanus* (Formosan land-locked salmon)	(Gwo et al., 1999a)	2	Canonical	Canonical	External- spawning	(Healey et al., 2001)
Vertebrata; Gnathostomata; Teleostomi; Euteleostomi; Actinopterygii; Actinopteri; Neopterygii; Teleostei; Osteoglossocephalai; Clupeocephala; Euteleosteomorpha; Protacanthopterygii; Salmoniformes; Salmonidae; Salmoninae; Oncorhynchus; Oncorhynchus masou	*Oncorhynchus masou formosanus* (Formosan landlocked salmon)	(Gwo et al., 1996) (Markova and Zhivkova, 2003b)	2	Canonical	Canonical	Internal- insemination	(Gwo et al., 1999b)
Vertebrata; Gnathostomata; Teleostomi; Euteleostomi; Actinopterygii; Actinopteri; Neopterygii; Teleostei; Osteoglossocephalai; Clupeocephala; Euteleosteomorpha; Protacanthopterygii; Salmoniformes; Salmonidae; Salmoninae; Brachymystax	*Brachymystax lenok Tsinlingensis* (Tsinling lenok trout)	(Guo et al., 2016)	2	Canonical	Canonical	External- spawning	(Yoon et al., 2015)
Vertebrata; Gnathostomata; Teleostomi; Euteleostomi; Actinopterygii; Actinopteri; Neopterygii; Teleostei; Osteoglossocephalai; Clupeocephala; Euteleosteomorpha; Protacanthopterygii; Salmoniformes; Salmonidae; Salmoninae; Oncorhynchus	*Oncorhynchus mykiss* (Rainbow trout)	(Markova and Zhivkova, 2003a)	2	Canonical	Canonical	External- spawning	(Sakamoto et al., 1999)
Vertebrata; Gnathostomata; Teleostomi; Euteleostomi; Actinopterygii; Actinopteri; Neopterygii; Teleostei; Osteoglossocephalai; Clupeocephala; Euteleosteomorpha; Protacanthopterygii; Salmoniformes; Salmonidae; Salmoninae; Salmo	*Salmo salar* (Atlantic salmon)	(Figueroa et al., 2017)	2	Canonical	Canonical	External- spawning	(Fleming, 1996)
Vertebrata; Gnathostomata; Teleostomi; Euteleostomi; Actinopterygii; Actinopteri; Neopterygii; Teleostei; Osteoglossocephalai; Clupeocephala; Euteleosteomorpha; Stomiati; Osmeriformes; Plecoglossidae; Plecoglossus	*Plecoglossus altivelis* (Ayu)	(Ohta et al., 1993)	2	Canonical	Canonical	External- spawning	(Otake et al., 2002)
Vertebrata; Gnathostomata; Teleostomi; Euteleostomi; Actinopterygii; Actinopteri; Neopterygii; Teleostei; Osteoglossocephalai; Clupeocephala; Euteleosteomorpha; Stomiati; Osmeriformes; Plecoglossidae; Plecoglossus	*Mallotus villosus* (Newfoundland capelin)	(Beirão et al., 2015)	2	Canonical	Canonical	External - spawning	(Carscadden et al., 1997)
Vertebrata; Gnathostomata; Teleostomi; Euteleostomi; Actinopterygii; Actinopteri; Neopterygii; Teleostei;	*Osmerus eperlanus*	(Kowalski et al., 2006)	2	Canonical	Canonical	External spawning	(Lyle and Maitland, 1997)

Osteoglossocephalai; Clupeocephala; Euteleosteomorpha; Stomiati; Osmeriformes; Osmeridae; Osmerinae; Osmerus								
Vertebrata; Gnathostomata; Teleostomi; Euteleostomi; Actinopterygii; Actinopteri; Neopterygii; Teleostei; Osteoglossocephalai; Clupeocephala; Euteleosteomorpha; Stomiati; Stomiiformes; Sternoptychidae; Sternoptychinae; Argyropelecus	*Argyropelecus gigas*	(Jamieson and Mattel, 2009)	2	Canonical PC is modified by extension; has microtubular prolongations	Canonical	External spawning (Determined based on Argyropelecus)	(Badcock et al., 1984) By Argyropelecus hemigymnus
Gnathostomata; Teleostomi; Euteleostomi; Actinopterygii; Actinopteri; Neopterygii; Teleostei; Osteoglossocephalai; Clupeocephala; Euteleosteomorpha; Stomiati; Stomiiformes	*Sternoptychidae*	(Jamieson, 1991)	2	Canonical	Canonical	External spawning	(Almeida and Rossi-Wongtschowski, 2007)
Gnathostomata; Teleostomi; Euteleostomi; Actinopterygii; Actinopteri; Neopterygii; Teleostei; Osteoglossocephalai; Clupeocephala; Euteleosteomorpha; Neoteleostei; Eurypterygia; Aulopa	*Aulopiformes*	(Jamieson, 1991)	2	Canonical	Canonical	External spawning	(Parenti et al., 2015)
Gnathostomata; Teleostomi; Euteleostomi; Actinopterygii; Actinopteri; Neopterygii; Teleostei; Osteoglossocephalai; Clupeocephala; Euteleosteomorpha; Neoteleostei; Eurypterygia; Ctenosquamata; Acanthomorphata; Paracanthopterygii; Zeiogadaria; Gadariae; Gadiformes; Gadoidei	*Merlucciidae*	(Jamieson, 1991)	2	Canonical	Canonical	External spawning	(Bustos et al., 2007)
Gnathostomata; Teleostomi; Euteleostomi; Actinopterygii; Actinopteri; Neopterygii; Teleostei; Osteoglossocephalai; Clupeocephala; Euteleosteomorpha; Neoteleostei; Eurypterygia; Ctenosquamata; Acanthomorphata; Euacanthomorphacea; Percomorphaceae; Ophidiaria; Ophidiiformes; Ophidioidei; Ophidiidae; Neobythitinae; Lamprogrammus	*Lamprogrammus exutus*	(Jamieson, 1991)	2	Canonical	Canonical	External spawning	(Mann et al., 1997)
Gnathostomata; Teleostomi; Euteleostomi; Actinopterygii; Actinopteri; Neopterygii; Teleostei; Osteoglossocephalai; Clupeocephala; Euteleosteomorpha; Neoteleostei; Eurypterygia; Ctenosquamata; Acanthomorphata; Euacanthomorphacea; Percomorphaceae; Batrachoidaria; Batrachoidiformes; Batrachoididae; Batrachoidinae	*Opsanus*	(Jamieson, 1991)	2	Canonical	Canonical	External spawning	(Barimo et al., 2007)
Gnathostomata; Teleostomi; Euteleostomi; Actinopterygii; Actinopteri; Neopterygii; Teleostei; Osteoglossocephalai; Clupeocephala; Euteleosteomorpha; Neoteleostei; Eurypterygia; Ctenosquamata; Acanthomorphata; Euacanthomorphacea; Holocentrimorphaceae; Holocentriformes; Holocentridae; Holocentrinae	*Holocentrus*	(Jamieson, 1991)	2	Canonical	Canonical	External spawning	(Shinozaki-Mendes et al., 2007)
Gnathostomata; Teleostomi; Euteleostomi; Actinopterygii; Actinopteri; Neopterygii; Teleostei; Osteoglossocephalai; Clupeocephala; Euteleosteomorpha; Neoteleostei; Eurypterygia; Ctenosquamata; Acanthomorphata; Euacanthomorphacea; Percomorphaceae; Anabantaria; Synbranchiformes; Synbranchoidei; Synbranchidae; Synbranchus	*Synbranchus marmoratus*	(Jamieson, 1991)	2	Canonical	Canonical	External spawning	(Lo Nostro et al., 2003)
Gnathostomata; Teleostomi; Euteleostomi; Actinopterygii; Actinopteri; Neopterygii; Teleostei; Osteoglossocephalai; Clupeocephala; Euteleosteomorpha; Neoteleostei;	*Hexagrammos agrammus*<br>*Hexagrammos octogrammus*	(Jamieson, 1991)	2	Canonical	Canonical	External spawning	(Munehara et al., 2000)

Eurypterygia; Ctenosquamata; Acanthomorphata; Euacanthomorphacea; Percomorphaceae; Eupercaria; Perciformes; Cottioidei; Hexagrammales	*Hexagrammos otakii*						
Gnathostomata; Teleostomi; Euteleostomi; Actinopterygii; Actinopteri; Neopterygii; Teleostei; Osteoglossocephalai; Clupeocephala; Euteleosteomorpha; Neoteleostei; Eurypterygia; Ctenosquamata; Acanthomorphata; Euacanthomorphacea; Percomorphaceae; Eupercaria; Perciformes; Cottioidei; Cottales; Cottidae; Alcichthys	*Alcichthys alcicornis*	(Jamieson, 1991)	2	Canonical	Canonical	External spawning	(Munehara, 1988)
Gnathostomata; Teleostomi; Euteleostomi; Actinopterygii; Actinopteri; Neopterygii; Teleostei; Osteoglossocephalai; Clupeocephala; Euteleosteomorpha; Neoteleostei; Eurypterygia; Ctenosquamata; Acanthomorphata; Euacanthomorphacea; Percomorphaceae; Eupercaria; Eupercaria incertae sedis	*Moronidae*	(Jamieson, 1991)	2	Canonical	Canonical	External spawning	(Martins et al., 2012)
Gnathostomata; Teleostomi; Euteleostomi; Actinopterygii; Actinopteri; Neopterygii; Teleostei; Osteoglossocephalai; Clupeocephala; Euteleosteomorpha; Neoteleostei; Eurypterygia; Ctenosquamata; Acanthomorphata; Euacanthomorphacea; Percomorphaceae; Eupercaria; Spariformes	*Pagrus major*<br>*Lagodon rhomboides*<br>*Acanthopagrus australis*<br>*Boops boops*<br>*Dilpodus sargus*	(Jamieson, 1991)	2	Canonical	Canonical	External spawning	(Matsuyama et al., 1988)
Gnathostomata; Teleostomi; Euteleostomi; Actinopterygii; Actinopteri; Neopterygii; Teleostei; Osteoglossocephalai; Clupeocephala; Euteleosteomorpha; Neoteleostei; Eurypterygia; Ctenosquamata; Acanthomorphata; Euacanthomorphacea; Percomorphaceae; Eupercaria; Labriformes	*Labridae*<br>*Thalassoma pavo*<br>*Thalassoma bifasciatum*<br>*Symphodus ocellatus*<br>*Lahnolaimus maximus*	(Jamieson, 1991)	2	Canonical	Canonical	External spawning	(Feddern, 1965)
Gnathostomata; Teleostomi; Euteleostomi; Actinopterygii; Actinopteri; Neopterygii; Teleostei; Osteoglossocephalai; Clupeocephala; Euteleosteomorpha; Neoteleostei; Eurypterygia; Ctenosquamata; Acanthomorphata; Euacanthomorphacea; Percomorphaceae; Eupercaria; Perciformes; Cottioidei; Zoarcales; Zoarcidae; Zoarcinae; Zoarces	*Zoarces elongatus*	(Jamieson, 1991)	2	Canonical	Canonical	External spawning	(De Lima and Araujo-Lima, 2004)
Gnathostomata; Teleostomi; Euteleostomi; Actinopterygii; Actinopteri; Neopterygii; Teleostei; Osteoglossocephalai; Clupeocephala; Euteleosteomorpha; Neoteleostei; Eurypterygia; Ctenosquamata; Acanthomorphata; Euacanthomorphacea; Percomorphaceae; Eupercaria; Perciformes; Cottioidei; Zoarcales; Anarhichadidae; Anarhichas	*Anarhichas lupus*	(Jamieson, 1991)	2	Canonical	Canonical	External spawning	(Johannessen et al., 1993)
Gnathostomata; Teleostomi; Euteleostomi; Actinopterygii; Actinopteri; Neopterygii; Teleostei; Osteoglossocephalai; Clupeocephala; Euteleosteomorpha; Neoteleostei; Eurypterygia; Ctenosquamata; Acanthomorphata; Euacanthomorphacea; Percomorphaceae; Eupercaria; Perciformes; Cottioidei; Cottales; Trichodontidae; Arctoscopus	*Arctoscopus japonicus*	(Jamieson, 1991)	2	Canonical	Canonical	External spawning	(Lee et al., 2006)
Gnathostomata; Teleostomi; Euteleostomi; Actinopterygii; Actinopteri; Neopterygii; Teleostei; Osteoglossocephalai; Clupeocephala; Euteleosteomorpha; Neoteleostei; Eurypterygia; Ctenosquamata; Acanthomorphata; Euacanthomorphacea; Percomorphaceae; Eupercaria; Uranoscopiformes; Pinguipedidae	*Parapercis*	(Jamieson, 1991)	2	Canonical	Canonical	External spawning	(Pankhurst and Kime, 1991)
Gnathostomata; Teleostomi; Euteleostomi; Actinopterygii; Actinopteri; Neopterygii; Teleostei; Osteoglossocephalai; Clupeocephala; Euteleosteomorpha; Neoteleostei; Eurypterygia; Ctenosquamata; Acanthomorphata; Euacanthomorphacea; Percomorphaceae; Eupercaria; Uranoscopiformes; Ammodytidae; Ammodytes	*Ammodytes personatus*	(Jamieson, 1991)	2	Canonical	Canonical	External spawning	(KIM et al., 1999)
Gnathostomata; Teleostomi; Euteleostomi; Actinopterygii; Actinopteri; Neopterygii; Teleostei; Osteoglossocephalai;	*Uranoscopus scaber*	(Jamieson, 1991)	2	Canonical	Canonical	External spawning	(Ak et al., 2011)

Clupeocephala; Euteleosteomorpha; Neoteleostei; Eurypterygia; Ctenosquamata; Acanthomorphata; Euacanthomorphacea; Percomorphaceae; Eupercaria; Uranoscopiformes							
Gnathostomata; Teleostomi; Euteleostomi; Actinopterygii; Actinopteri; Neopterygii; Teleostei; Osteoglossocephalai; Clupeocephala; Euteleosteomorpha; Neoteleostei; Eurypterygia; Ctenosquamata; Acanthomorphata; Euacanthomorphacea; Percomorphaceae; Eupercaria; Perciformes; Percoidei	*Trachinidae*	(Jamieson, 1991)	2	Canonical	Canonical	External spawning	(Hamed and Chakroun-Marzouk, 2017)
Gnathostomata; Teleostomi;Euteleostomi; Actinopterygii; Actinopteri; Neopterygii; Teleostei; Osteoglossocephalai; Clupeocephala; Euteleosteomorpha; Neoteleostei; Eurypterygia; Ctenosquamata; Acanthomorphata; Euacanthomorphacea; Percomorphaceae; Eupercaria; Tetraodontiformes; Tetraodontoidei; Tetradontoidea; Tetraodontidae; Tetractenos	*Tetractenos hamiltoni*	(Jamieson, 1991)	2	Canonical	Canonical	External spawning	(Piah and Bucher, 2014)
Gnathostomata; Teleostomi; Euteleostomi; Actinopterygii; Actinopteri; Neopterygii; Teleostei; Osteoglossocephalai; Clupeocephala; Euteleosteomorpha; Neoteleostei; Eurypterygia; Ctenosquamata; Acanthomorphata; Euacanthomorphacea; Percomorphaceae; Eupercaria; Centrarchiformes; Percichthyoidei; Percichthyidae; Nannoperca	*Nannoperca oxleyana*	(Jamieson, 1991)	2	Canonical	Canonical	External spawning	(Knight et al., 2007)
Gnathostomata; Teleostomi; Euteleostomi; Actinopterygii; Actinopteri; Neopterygii; Teleostei; Osteoglossocephalai; Clupeocephala; Euteleosteomorpha; Neoteleostei; Eurypterygia; Ctenosquamata; Acanthomorphata; Euacanthomorphacea; Percomorphaceae; Eupercaria; Lophiiformes; Ceratioidei; Neoceratiidae; Neoceratias	*Neoceratias spinifer*	(Jamieson, 1991)	2	Canonical	Canonical	External spawning	(Pietsch, 2005)
Gnathostomata; Teleostomi; Euteleostomi; Actinopterygii; Actinopteri; Neopterygii; Teleostei; Osteoglossocephalai; Clupeocephala; Euteleosteomorpha; Neoteleostei; Eurypterygia; Ctenosquamata; Acanthomorphata; Euacanthomorphacea; Percomorphaceae; Eupercaria; Tetraodontiformes; Balistoidea; Balistidae; Pseudobalistes	*Pseudobalistes fuscus*	(Jamieson, 1991)	2	Canonical	Canonical	External spawning	(Gladstone, 1994)
Gnathostomata; Teleostomi; Euteleostomi; Actinopterygii; Actinopteri; Neopterygii; Teleostei; Osteoglossocephalai; Clupeocephala; Euteleosteomorpha; Neoteleostei; Eurypterygia; Ctenosquamata; Acanthomorphata; Euacanthomorphacea; Percomorphaceae; Eupercaria; Perciformes; Serranoidei; Serranidae; Epinephelinae; Epinephelini; Plectropomus	*Plectropomus leopardus*	(Jamieson, 1991)	2	Canonical	Canonical	External spawning	(Zeller, 1998)
Vertebrata; Actinopterygii; Teleostei ; Osteoglossocephalai; Clupeocephala; Euteleosteomorpha; Neoteleostei; Eurypterygia; Ctenosquamata; Acanthomorphata; Euacanthomorphacea; Percomorphaceae; Eupercaria; Eupercaria incertae sedis; Sciaenidae; Miichthys	*Miichthys miiuy*	(Miao et al., 2013)	2	Canonical	Canonical	External- spawning	(Yoon et al., 2006)
Vertebrata; Gnathostomata; Teleostomi; Euteleostomi; Actinopterygii; Actinopteri; Neopterygii; Teleostei; Osteoglossocephalai; Clupeocephala; Euteleosteomorpha; Neoteleostei; Eurypterygia; Ctenosquamata; Acanthomorphata; Euacanthomorphacea; Percomorphaceae; Eupercaria; Eupercaria incertae sedis; Sciaenidae; Paralonchurus	*Paralonchurus brasiliensis* (Banded croaker)	(Gusmao-Pompiani et al., 2005)	2	Canonical	Canonical	External spawning	(Costa et al., 2015)
Vertebrata; Gnathostomata; Teleostomi; Euteleostomi; Actinopterygii; Actinopteri; Neopterygii; Teleostei; Osteoglossocephalai; Clupeocephala; Euteleosteomorpha; Neoteleostei; Eurypterygia; Ctenosquamata; Acanthomorphata; Euacanthomorphacea; Percomorphaceae; Eupercaria; Spariformes; Sparidae; Acanthopagrus	*Acanthopagrus latus*	(Gwo, 1995)	2	Canonical	Canonical	External -spawning	(Alex Hesp et al., 2004)
	Acanthopagrus australis	(Gwo et al., 2005)	2	Canonical	Canonical	External -spawning	(Alex Hesp et al., 2004)
Vertebrata; Gnathostomata; Teleostomi; Euteleostomi; Actinopterygii; Actinopteri; Neopterygii; Teleostei; Osteoglossocephalai; Clupeocephala; Euteleosteomorpha; Neoteleostei; Eurypterygia; Ctenosquamata;	*Epinephelus malabaricus* (Malabar grouper)	(GWO et al., 1994) (Jamieson, 1991)	2	Canonical	Canonical	External spawning	(Gaspare and Bryceson, 2013)

Acanthomorphata; Euacanthomorphacea; Percomorphaceae; Eupercaria; Perciformes; Serranoidei; Serranidae; Epinephelinae; Epinephelini; Epinephelus							
Vertebrata; Gnathostomata; Teleostomi; Euteleostomi; Actinopterygii; Actinopteri; Neopterygii; Teleostei; Osteoglossocephalai; Clupeocephala; Euteleosteomorpha; Neoteleostei; Eurypterygia; Ctenosquamata; Acanthomorphata; Euacanthomorphacea; Percomorphaceae; Eupercaria; Eupercaria incertae sedis; Sciaenidae; Larimus	*Larimus breviceps* (Shorthead drum)	(Gusmao-Pompiani et al., 2005)	2	Canonical	Canonical	External spawning	(Santos et al., 2021)
Vertebrata; Gnathostomata; Teleostomi; Euteleostomi; Actinopterygii; Actinopteri; Neopterygii; Teleostei; Osteoglossocephalai; Clupeocephala; Euteleosteomorpha; Neoteleostei; Eurypterygia; Ctenosquamata; Acanthomorphata; Euacanthomorphacea; Percomorphaceae; Eupercaria; Eupercaria incertae sedis; Sciaenidae; Cynoscion	*Cynoscion striatus* (South American striped weakfish)	(Gusmao-Pompiani et al., 2005)	2	Canonical	Canonical	External spawning	(Militelli and Macchi, 2006)
Vertebrata; Gnathostomata; Teleostomi; Euteleostomi; Actinopterygii; Actinopteri; Neopterygii; Teleostei; Osteoglossocephalai; Clupeocephala; Euteleosteomorpha; Neoteleostei; Eurypterygia; Ctenosquamata; Acanthomorphata; Euacanthomorphacea; Percomorphaceae; Eupercaria; Eupercaria incertae sedis; Sciaenidae; Micropogonias	*Micropogonias furnieri* (Whitemouth croaker)	(Gusmao-Pompiani et al., 2005)	2	Canonical	Canonical	External spawning	(Acha et al., 1999)
Vertebrata; Gnathostomata; Teleostomi; Euteleostomi; Actinopterygii; Actinopteri; Neopterygii; Teleostei; Osteoglossocephalai; Clupeocephala; Euteleosteomorpha; Neoteleostei; Eurypterygia; Ctenosquamata; Acanthomorphata; Euacanthomorphacea; Percomorphaceae; Eupercaria; Eupercaria incertae sedis; Sciaenidae; Menticirrhus	*Menticirrhus americanus* (Southern kingcroaker)	(Gusmao-Pompiani et al., 2005)	2	Canonical	Canonical	External spawning	(Harding and Chittenden, 1987)
Vertebrata; Gnathostomata; Teleostomi; Euteleostomi; Actinopterygii; Actinopteri; Neopterygii; Teleostei; Osteoglossocephalai; Clupeocephala; Euteleosteomorpha; Neoteleostei; Eurypterygia; Ctenosquamata; Acanthomorphata; Euacanthomorphacea; Percomorphaceae; Eupercaria; Eupercaria incertae sedis; Sciaenidae; Umbrina	*Umbrina coroides* (Shi drum)	(Gusmao-Pompiani et al., 2005)	2	Canonical	Canonical	External spawning	{Breder, 1966 #390} By Fishbase
Vertebrata; Gnathostomata; Teleostomi; Euteleostomi; Actinopterygii; Actinopteri; Neopterygii; Teleostei; Osteoglossocephalai; Clupeocephala; Euteleosteomorpha; Neoteleostei; Eurypterygia; Ctenosquamata; Acanthomorphata; Euacanthomorphacea; Percomorphaceae; Eupercaria; Eupercaria incertae sedis; Sciaenidae; Stellifer	*Stellifer rastrifer* (rake stardrum)	(Gusmao-Pompiani et al., 2005)	2	Canonical	Canonical	External spawning	(Camargo and Isaac, 2005)
Vertebrata; Gnathostomata; Teleostomi; Euteleostomi; Actinopterygii; Actinopteri; Neopterygii; Teleostei; Osteoglossocephalai; Clupeocephala; Euteleosteomorpha; Neoteleostei; Eurypterygia; Ctenosquamata; Acanthomorphata; Euacanthomorphacea; Percomorphaceae; Eupercaria; Eupercaria incertae sedis; Sciaenidae; Plagioscion	*Plagioscion squamosissimus* (South American silver croaker)	(Gusmao-Pompiani et al., 2005)	2	Canonical	Canonical	External spawning	(Bialetzki et al., 2004)
Vertebrata; Gnathostomata; Teleostomi; Euteleostomi; Actinopterygii; Actinopteri; Neopterygii; Teleostei; Osteoglossocephalai; Clupeocephala; Euteleosteomorpha; Neoteleostei; Eurypterygia; Ctenosquamata; Acanthomorphata; Euacanthomorphacea; Percomorphaceae; Eupercaria; Eupercaria incertae sedis; Siganidae; Siganus	*Siganus fuscescens* (mottled spinefoot)<br>*Siganus rivulatus*	(Gwo et al., 2004b)	2	Canonical	Canonical	External spawning	(Hara et al., 1986)
Vertebrata; Gnathostomata; Teleostomi; Euteleostomi; Actinopterygii; Actinopteri; Neopterygii; Teleostei; Osteoglossocephalai; Clupeocephala; Euteleosteomorpha; Neoteleostei; Eurypterygia; Ctenosquamata; Acanthomorphata; Euacanthomorphacea; Percomorphaceae; Eupercaria; Perciformes; Serranoidei; Serranidae; Epinephelinae; Epinephelini; Epinephelus	*Epinephelus bruneus* (Longtooth grouper)	(Lim and Le, 2013) (Kim et al., 2013)	2	Canonical	Canonical	External spawning	(Song et al., 2005)
Vertebrata; Gnathostomata; Teleostomi; Euteleostomi; Actinopterygii; Actinopteri; Neopterygii; Teleostei; Osteoglossocephalai; Clupeocephala; Euteleosteomorpha; Neoteleostei; Eurypterygia; Ctenosquamata;	*Sander lucioperca* (Pike-perch)	(Lahnsteiner and Mansour, 2004) (Křišťan et al., 2014)	2	Canonical	Canonical	External spawning	(Lappalainen et al., 2003)

Acanthomorphata; Euacanthomorphacea; Percomorphaceae; Eupercaria; Perciformes; Percoidei; Percidae; Luciopercinae; Sander							
Vertebrata; Gnathostomata; Teleostomi; Euteleostomi; Actinopterygii; Actinopteri; Neopterygii; Teleostei; Osteoglossocephalai; Clupeocephala; Euteleosteomorpha; Neoteleostei; Eurypterygia; Ctenosquamata; Acanthomorphata; Euacanthomorphacea; Percomorphaceae; Eupercaria; Centrarchiformes; Centrarchoidei; Sinipercidae; Siniperca	*Siniperca chuatsi* (Mandarin fish)	(Luo et al., 2011)	2	Canonical	Canonical	External spawning	(Doi et al., 2004)
	Siniperca kneri (Big-eye mandarin fish)	(Luo et al., 2011)	2	Canonical	Canonical	External spawning	(Zhao et al., 2019)
	Siniperca scherzeri (Leopard mandarin fish)	(Luo et al., 2011)	2	Canonical	Canonical	External spawning	(Zhang et al., 2009)
Vertebrata; Gnathostomata; Teleostomi; Euteleostomi; Actinopterygii; Actinopteri; Neopterygii; Teleostei; Osteoglossocephalai; Clupeocephala; Euteleosteomorpha; Neoteleostei; Eurypterygia; Ctenosquamata; Acanthomorphata; Euacanthomorphacea; Percomorphaceae; Eupercaria; Eupercaria incertae sedis; Scatophagidae; Scatophagus	*Scatophagus argus*	(Madhavi et al., 2015)	2	Canonical	Canonical	External spawning	(Cai et al., 2010)
Vertebrata; Gnathostomata; Teleostomi; Euteleostomi; Actinopterygii; Actinopteri; Neopterygii; Teleostei; Osteoglossocephalai; Clupeocephala; Euteleosteomorpha; Neoteleostei; Eurypterygia; Ctenosquamata; Acanthomorphata; Euacanthomorphacea; Percomorphaceae; Eupercaria; Perciformes; Percoidei; Percidae; Percinae; Perca	*Perca Fluviatilis* (Eurasian perch)	(Hatef et al., 2013)	2	Canonical	Canonical	External spawning	(Snickars et al., 2010)
Vertebrata; Gnathostomata; Teleostomi; Euteleostomi; Actinopterygii; Actinopteri; Neopterygii; Teleostei; Osteoglossocephalai; Clupeocephala; Euteleosteomorpha; Neoteleostei; Eurypterygia; Ctenosquamata; Acanthomorphata; Euacanthomorphacea; Percomorphaceae; Eupercaria; Perciformes; Scorpaenoidei; Scorpaenidae; Scorpaeninae; Parascorpaena	*Parascorpaena picta*	(Pavlov and Emel'yanova, 2018)	2	Canonical	Canonical	Internal insemination	(Natal'ya and Pavlov, 2017)
Vertebrata; Gnathostomata; Teleostomi; Euteleostomi; Actinopterygii; Actinopteri; Neopterygii; Teleostei; Osteoglossocephalai; Clupeocephala; Euteleosteomorpha; Neoteleostei; Eurypterygia; Ctenosquamata; Acanthomorphata; Euacanthomorphacea; Percomorphaceae; Eupercaria; Perciformes; Cottioidei; Cottales; Cottidae; Cottus	*Cottus hangiongensis*	(Quinitio and Takahashi, 1992)	2	Canonical	Canonical	External spawning	(Goto, 1988)
Vertebrata; Gnathostomata; Teleostomi; Euteleostomi; Actinopterygii; Actinopteri; Neopterygii; Teleostei; Osteoglossocephalai; Clupeocephala; Euteleosteomorpha; Neoteleostei; Eurypterygia; Ctenosquamata; Acanthomorphata; Euacanthomorphacea; Percomorphaceae; Eupercaria; Tetraodontiformes; Tetraodontoidei; Tetradontoidea; Tetraodontidae; Takifugu	*Takifugu niphobles* (pufferfish)	(Morisawa, 2001)	2	Canonical	Canonical	External spawning	(Gallego et al., 2013)
	Takifugu paradilis						
	Takifugu peocilonotus						
	Takifugu porphyreus						
	Takifugu radiatus						
	Takifugu rubruipes						
	Takifugu xanthopterus						
Vertebrata; Gnathostomata; Teleostomi; Euteleostomi; Actinopterygii; Actinopteri; Neopterygii; Teleostei; Osteoglossocephalai; Clupeocephala; Euteleosteomorpha; Neoteleostei; Eurypterygia; Ctenosquamata; Acanthomorphata; Euacanthomorphacea; Percomorphaceae; Eupercaria; Spariformes; Sparidae; Pagellus	*Pagellus bogaraveo* (Blackspot seabream)	(Maricchiolo et al., 2010)	2	Canonical	Canonical	External spawning	(Micale et al., 2002)
Gnathostomata; Teleostomi; Euteleostomi; Actinopterygii; Actinopteri; Neopterygii; Teleostei; Osteoglossocephalai; Clupeocephala; Euteleosteomorpha; Neoteleostei; Eurypterygia; Ctenosquamata; Acanthomorphata; Euacanthomorphacea; Percomorphaceae; Eupercaria; Perciformes; Serranoidei; Serranidae; Serraninae	*Serranus atricauda*	(Jamieson, 1991)	2	Canonical	Canonical	External spawning	(Neves et al., 2014)
	Serranus cabrilla						
	Serranus scriba						
Gnathostomata; Teleostomi; Euteleostomi; Actinopterygii; Actinopteri; Neopterygii; Teleostei; Osteoglossocephalai; Clupeocephala; Euteleosteomorpha; Neoteleostei; Eurypterygia; Ctenosquamata; Acanthomorphata; Euacanthomorphacea; Percomorphaceae; Ovalentaria;	*Jenynsia lineata*	(Jamieson, 1991)	2	Canonical	Canonical	internal	{Wischnath, 1993 #389} (Barimo et al., 2007) By fishbase

Atherinomorphae; Cyprinodontiformes; Cyprinodontoidei; Anablepidae; Jenynsiinae; Jenynsia							
Gnathostomata; Teleostomi; Euteleostomi; Actinopterygii; Actinopteri; Neopterygii; Teleostei; Osteoglossocephalai; Clupeocephala; Euteleosteomorpha; Neoteleostei; Eurypterygia; Ctenosquamata; Acanthomorphata; Euacanthomorphacea; Percomorphaceae; Ovalentaria; Atherinomorphae; Cyprinodontiformes; Aplocheiloidei; Nothobranchiidae; Fundulopanchax	*Fundulopanchax gardneri*	(Jamieson, 1991)	2	Canonical	Canonical	external spawning	(Furness et al., 2015)
Gnathostomata; Teleostomi; Euteleostomi; Actinopterygii; Actinopteri; Neopterygii; Teleostei; Osteoglossocephalai; Clupeocephala; Euteleosteomorpha; Neoteleostei; Eurypterygia; Ctenosquamata; Acanthomorphata; Euacanthomorphacea; Percomorphaceae; Ovalentaria; Ovalentaria incertae sedis; Embiotocidae; Cymatogaster	*Cymatogaster aggregata*	(Jamieson, 1991)	2	Canonical	Canonical	External spawning	(Gordon, 1965)
Gnathostomata; Teleostomi; Euteleostomi; Actinopterygii; Actinopteri; Neopterygii; Teleostei; Osteoglossocephalai; Clupeocephala; Euteleosteomorpha; Neoteleostei; Eurypterygia; Ctenosquamata; Acanthomorphata; Euacanthomorphacea; Percomorphaceae; Ovalentaria; Ovalentaria incertae sedis; Pomacentridae; Chromis	*Chromis dispilus*	(Jamieson, 1991)	2	Canonical	Canonical	External spawning	(Tzioumis and Kingsford, 1995)
Gnathostomata; Teleostomi; Euteleostomi; Actinopterygii; Actinopteri; Neopterygii; Teleostei; Osteoglossocephalai; Clupeocephala; Euteleosteomorpha; Neoteleostei; Eurypterygia; Ctenosquamata; Acanthomorphata; Euacanthomorphacea; Percomorphaceae; Ovalentaria; Blenniimorphae; Blenniiformes;	*Parablennius parvicornis*<br>*Parablennius tentacularis*<br>*Entromacrodus straitus*<br>*Blenniella bilitonenis*<br>*Rhadoblennous ellipes*	(Jamieson, 1991)	2	Canonical	Canonical	External spawning	(Cody, 1993)
Gnathostomata; Teleostomi; Euteleostomi; Actinopterygii; Actinopteri; Neopterygii; Teleostei; Osteoglossocephalai; Clupeocephala; Euteleosteomorpha; Neoteleostei; Eurypterygia; Ctenosquamata; Acanthomorphata; Euacanthomorphacea; Percomorphaceae; Ovalentaria; Atherinomorphae; Cyprinodontiformes; Cyprinodontoidei; Fundulidae; Fundulus	*Fundulus heteroclitus*	(Jamieson, 1991)	2	Canonical	Canonical	External spawning	(Taylor et al., 1979)
Gnathostomata; Teleostomi; Euteleostomi; Actinopterygii; Actinopteri; Neopterygii; Teleostei; Osteoglossocephalai; Clupeocephala; Euteleosteomorpha; Neoteleostei; Eurypterygia; Ctenosquamata; Acanthomorphata; Euacanthomorphacea; Percomorphaceae; Ovalentaria; Atherinomorphae; Cyprinodontiformes; Aplocheiloidei; Nothobranchiidae; Fundulopanchax	*Aphyosemion gardneri*	(Jamieson, 1991)	2	Canonical	Canonical	External spawning	(Kroll, 1984)
Gnathostomata; Teleostomi; Euteleostomi; Actinopterygii; Actinopteri; Neopterygii; Teleostei; Osteoglossocephalai; Clupeocephala; Euteleosteomorpha; Neoteleostei; Eurypterygia; Ctenosquamata; Acanthomorphata; Euacanthomorphacea; Percomorphaceae; Ovalentaria; Atherinomorphae; Beloniformes; Belonoidei; Hemiramphidae; Arrhamphus	*Arramphus sclerolepis*	(Jamieson, 1991)	2	Canonical	Canonical	External spawning	(Gratani et al., 2016)
Gnathostomata; Teleostomi; Euteleostomi; Actinopterygii; Actinopteri; Neopterygii; Teleostei; Osteoglossocephalai; Clupeocephala; Euteleosteomorpha; Neoteleostei; Eurypterygia; Ctenosquamata; Acanthomorphata; Euacanthomorphacea; Percomorphaceae; Ovalentaria; Atherinomorphae; Atheriniformes; Atherinidae; Atherininae; Craterocephalus	Craterocephalus marjoriae<br>*Craterocehphalus stercusmuscarum*<br>*Craterocehphalus helenae*<br>*Craterocehphalus stramineus*	(Jamieson, 1991)	2	Canonical	Canonical	External spawning	(MILTON and ARTHINGTON, 1983)
Gnathostomata; Teleostomi; Euteleostomi; Actinopterygii; Actinopteri; Neopterygii; Teleostei; Osteoglossocephalai; Clupeocephala; Euteleosteomorpha; Neoteleostei;	*Pseudomugil mellis*<br>*Pseudomugil Signifer*	(Jamieson, 1991)	2	Canonical	Canonical	External spawning	(Semple, 1991)

Eurypterygia; Ctenosquamata; Acanthomorphata; Euacanthomorphacea; Percomorphaceae; Ovalentaria; Atherinomorphae; Atheriniformes; Pseudomugilidae; Pseudomugil	*Pseudomugil tenellus*						
Gnathostomata; Teleostomi; Euteleostomi; Actinopterygii; Actinopteri; Neopterygii; Teleostei; Osteoglossocephalai; Clupeocephala; Euteleosteomorpha; Neoteleostei; Eurypterygia; Ctenosquamata; Acanthomorphata; Euacanthomorphacea; Percomorphaceae; Ovalentaria; Atherinomorphae; Atheriniformes; Melanotaeniidae; Iriatherina	*Iriatherina werneri*	(Jamieson, 1991)	2	Canonical	Canonical	External spawning	(Herjayanto et al., 2016)
Gnathostomata; Teleostomi; Euteleostomi; Actinopterygii; Actinopteri; Neopterygii; Teleostei; Osteoglossocephalai; Clupeocephala; Euteleosteomorpha; Neoteleostei; Eurypterygia; Ctenosquamata; Acanthomorphata; Euacanthomorphacea; Percomorphaceae; Ovalentaria; Atherinomorphae; Atheriniformes; Melanotaeniidae; Melanotaenia	*Melanotaenia Maccullochi*<br>*Melanotaenia Duboulayi*	(Jamieson, 1991)	2	Canonical	Canonical	External spawning	(Oulton et al., 2013)
Gnathostomata; Teleostomi; Euteleostomi; Actinopterygii; Actinopteri; Neopterygii; Teleostei; Osteoglossocephalai; Clupeocephala; Euteleosteomorpha; Neoteleostei; Eurypterygia; Ctenosquamata; Acanthomorphata; Euacanthomorphacea; Percomorphaceae; Ovalentaria; Atherinomorphae; Atheriniformes; Melanotaeniidae;	*Melanotaenia*	(Jamieson, 1991)	2	Canonical	Canonical	External spawning	(Reid and Holdway, 1995)
Gnathostomata; Teleostomi; Euteleostomi; Actinopterygii; Actinopteri; Neopterygii; Teleostei; Osteoglossocephalai; Clupeocephala; Euteleosteomorpha; Neoteleostei; Eurypterygia; Ctenosquamata; Acanthomorphata; Euacanthomorphacea; Percomorphaceae; Ovalentaria; Blenniimorphae; Blenniiformes; Gobiesocoidei; Gobiesocidae; Gobiesocinae	*Lepadogaster lepadogaster*	(Jamieson, 1991)	2	Canonical	Canonical	External spawning	(Faria and Gonçalves, 2010)
Gnathostomata; Teleostomi; Euteleostomi; Actinopterygii; Actinopteri; Neopterygii; Teleostei; Osteoglossocephalai; Clupeocephala; Euteleosteomorpha; Neoteleostei; Eurypterygia; Ctenosquamata; Acanthomorphata; Euacanthomorphacea; Percomorphaceae; Ovalentaria; Mugilomorphae; Mugiliformes; Mugilidae; Chelon	*Liza dumerilii*	(Jamieson, 1991)	2	Canonical	Canonical	External spawning	(Van der Horst and Erasmus, 1981)
Gnathostomata; Teleostomi; Euteleostomi; Actinopterygii; Actinopteri; Neopterygii; Teleostei; Osteoglossocephalai; Clupeocephala; Euteleosteomorpha; Neoteleostei; Eurypterygia; Ctenosquamata; Acanthomorphata; Euacanthomorphacea; Percomorphaceae; Ovalentaria; Atherinomorphae; Cyprinodontiformes; Cyprinodontoidei; Poeciliidae; Poeciliinae; Poecilia	*Poecilia reticulata*	(Jamieson, 1991) (Billard et al., 1970)	1 (Confirmed)	Atypical triplets become occluded and is reduced to a remnant	Canonical	Internal insemination- no spermcasting	(Boschetto et al., 2011)
Gnathostomata; Teleostomi; Euteleostomi; Actinopterygii; Actinopteri; Neopterygii; Teleostei; Osteoglossocephalai; Clupeocephala; Euteleosteomorpha; Neoteleostei; Eurypterygia; Ctenosquamata; Acanthomorphata; Euacanthomorphacea; Percomorphaceae; Ovalentaria; Atherinomorphae; Cyprinodontiformes; Cyprinodontoidei; Poeciliidae; Poeciliinae; Poecilia	*Poecilia latipinna*	(Grier, 1973).	1 (Confirmed)	Atypical	Canonical	Internal insemination	(Boschetto et al., 2011)
Vertebrata; Gnathostomata; Teleostomi; Euteleostomi; Actinopterygii; Actinopteri; Neopterygii; Teleostei; Osteoglossocephalai; Clupeocephala; Euteleosteomorpha; Neoteleostei; Eurypterygia; Ctenosquamata; Acanthomorphata; Euacanthomorphacea; Percomorphaceae; Ovalentaria; Atherinomorphae; Atheriniformes; Atherinopsidae; Atherinopsinae; Odontesthes	*Odontesthes bonariensis*	(Gárriz and Miranda, 2013)	2	Canonica	Canonical	External- spawning	(Miranda et al., 2006)
Vertebrata; Gnathostomata; Teleostomi; Euteleostomi; Actinopterygii; Actinopteri; Neopterygii; Teleostei; Osteoglossocephalai; Clupeocephala; Euteleosteomorpha; Neoteleostei; Eurypterygia; Ctenosquamata; Acanthomorphata; Euacanthomorphacea; Percomorphaceae; Ovalentaria; Atherinomorphae; Cyprinodontiformes; Cyprinodontoidei; Poeciliidae; Poeciliinae; Gambusia	Gambusia affinis (Estern mosquitofish)	Spermiogenesis in the Teleost Gambusia affinis with Particular Reference to the Role Played by Microtubules H.J. Grier	1 (Confirmed)	Atypical " Proximal centriole (pc) is electron dense and no longer contains distinct tubules"	Canonical	Internal	(Zhu et al., 2015)

Vertebrata; Gnathostomata; Teleostomi; Euteleostomi; Actinopterygii; Actinopteri; Neopterygii; Teleostei; Osteoglossocephalai; Clupeocephala; Euteleosteomorpha; Neoteleostei; Eurypterygia; Ctenosquamata; Acanthomorphata; Euacanthomorphacea; Percomorphaceae; Ovalentaria; Cichlomorphae; Cichliformes; Cichlidae; African cichlids; Pseudocrenilabrinae; Oreochromini; Oreochromis	Oreochromis niloticus (Nile tilapia)	(Shalaby and Migeed, 2012)	2	Canonical	Canonical	External spawning	(LITTLE et al., 1993b)
Vertebrata; Gnathostomata; Teleostomi; Euteleostomi; Actinopterygii; Actinopteri; Neopterygii; Teleostei; Osteoglossocephalai; Clupeocephala; Euteleosteomorpha; Neoteleostei; Eurypterygia; Ctenosquamata; Acanthomorphata; Euacanthomorphacea; Percomorphaceae; Ovalentaria; Atherinomorphae; Cyprinodontiformes; Aplocheiloidei; Rivulidae; Melanorivulus	Melanorivulus punctatus	(Cassel et al., 2014)	2	Canonical	Canonical	External spawning	(Cassel et al., 2013)
Vertebrata; Gnathostomata; Teleostomi; Euteleostomi; Actinopterygii; Actinopteri; Neopterygii; Teleostei; Osteoglossocephalai; Clupeocephala; Euteleosteomorpha; Neoteleostei; Eurypterygia; Ctenosquamata; Acanthomorphata; Euacanthomorphacea; Percomorphaceae; Ovalentaria; Atherinomorphae; Beloniformes; Belonoidei; Zenarchopteridae; Hemirhamphodon	*Hemirhamphodon pogonognathus*	(Jamieson, 1989)	1 (Confirmed)	Undetected	Canonical	Internal- no spermcasting	(Jamieson and Grier, 1993)
Vertebrata; Gnathostomata; Teleostomi; Euteleostomi; Actinopterygii; Actinopteri; Neopterygii; Teleostei; Osteoglossocephalai; Clupeocephala; Euteleosteomorpha; Neoteleostei; Eurypterygia; Ctenosquamata; Acanthomorphata; Euacanthomorphacea; Percomorphaceae; Ovalentaria; Cichlomorphae; Cichliformes; Cichlidae; New World cichlids; Geophaginae; Geophagini; Satanoperca	*Satanoperca jurupari* (Demon eartheater)	(Matos et al., 2002)	2	Canonical	Canonical	External spawning	(Tavares-Dias et al., 2017)
Vertebrata; Gnathostomata; Teleostomi; Euteleostomi; Actinopterygii; Actinopteri; Neopterygii; Teleostei; Osteoglossocephalai; Clupeocephala; Euteleosteomorpha; Neoteleostei; Eurypterygia; Ctenosquamata; Acanthomorphata; Euacanthomorphacea; Percomorphaceae; Ovalentaria; Atherinomorphae; Cyprinodontiformes; Cyprinodontoidei; Anablepidae; Anablepinae; Anableps	*Anableps anableps*	(Greven and Schmahl, 2006) (Jamieson, 1991)	2	Canonical	Canonical	internal	(Turner, 1938)
Vertebrata; Gnathostomata; Teleostomi; Euteleostomi; Actinopterygii; Actinopteri; Neopterygii; Teleostei; Osteoglossocephalai; Clupeocephala; Euteleosteomorpha; Neoteleostei; Eurypterygia; Ctenosquamata; Acanthomorphata; Euacanthomorphacea; Percomorphaceae; Ovalentaria; Cichlomorphae; Cichliformes; Cichlidae; New World cichlids; Cichlinae; Cichlini; Cichla	*Cichla intermedia*	(Quagio-Grassiotto et al., 2003)	2	Canonical	Canonical	external	(Willis et al., 2007)
Vertebrata; Gnathostomata; Teleostomi; Euteleostomi; Actinopterygii; Actinopteri; Neopterygii; Teleostei; Osteoglossocephalai; Clupeocephala; Euteleosteomorpha; Neoteleostei; Eurypterygia; Ctenosquamata; Acanthomorphata; Euacanthomorphacea; Percomorphaceae; Ovalentaria; Mugilomorphae; Mugiliformes; Mugilidae; Chelon	*Chelon auratus*	(Bruslé, 1981)	2	Canonical	Canonical	External spawning	(Kesiktaş et al., 2020)
Vertebrata; Gnathostomata; Teleostomi; Euteleostomi; Actinopterygii; Actinopteri; Neopterygii; Teleostei; Osteoglossocephalai; Clupeocephala; Euteleosteomorpha; Neoteleostei; Eurypterygia; Ctenosquamata; Acanthomorphata; Euacanthomorphacea; Percomorphaceae; Ovalentaria; Atherinomorphae; Beloniformes; Adrianichthyoidei; Adrianichthyidae; Oryziinae; Oryzias	*Oryzias latipes*	(Grier, 1976)	2	Undetected	Canonical	External spawning	(Koger et al., 1999)
Vertebrata; Gnathostomata; Teleostomi; Euteleostomi; Actinopterygii; Actinopteri; Neopterygii; Teleostei; Osteoglossocephalai; Clupeocephala; Euteleosteomorpha; Neoteleostei; Eurypterygia; Ctenosquamata; Acanthomorphata; Euacanthomorphacea; Percomorphaceae; Ovalentaria; Atherinomorphae; Cyprinodontiformes; Cyprinodontoidei; Poeciliidae; Poeciliinae; Poecilia	*Lebistes reticulatus*	(Mattei and Boisson, 1966) (Grönberg and Telkkä, 1967) (Gronberg and Wartiovaara, 1972) (Asai, 1971)	unknown	PC is present in spermatid at stage that chromatid material has begun to condensate	DC is present in spermatid at stage that chromatid material has begun to condensate	External spawning	(Brewer, 1979)

nathostomata; Teleostomi; Euteleostomi; Actinopterygii; ctinopteri; Neopterygii; Teleostei; Osteoglossocephalai; lupeocephala; Euteleosteomorpha; Neoteleostei; urypterygia; Ctenosquamata; Acanthomorphata; uacanthomorphacea; Percomorphaceae; Ovalentaria; valentaria incertae sedis; Opistognathidae; Opistognathus	*Opistognathus whitehursti*	(Jamieson, 1991)	2	Canonical	Canonical	External spawning	(Thompson, 1974) (Demirel and Yüksek, 2013)
Gnathostomata; Teleostomi; Euteleostomi; Actinopterygii; Actinopteri; Neopterygii; Teleostei; Osteoglossocephalai; Clupeocephala; Euteleosteomorpha; Neoteleostei; Eurypterygia; Ctenosquamata; Acanthomorphata; Euacanthomorphacea; Percomorphaceae; Syngnathiaria; Syngnathiformes; Syngnathoidei; Syngnathidae; Syngnathinae	*Syngnathus acusimilis*	(Jamieson, 1991)	2	Canonical	Canonical	External spawning	(Kornienko, 2001)
	Syngnathus abaster						
Gnathostomata; Teleostomi; Euteleostomi; Actinopterygii; Actinopteri; Neopterygii; Teleostei; Osteoglossocephalai; Clupeocephala; Euteleosteomorpha; Neoteleostei; Eurypterygia; Ctenosquamata; Acanthomorphata; Euacanthomorphacea; Percomorphaceae; Syngnathiaria; Syngnathiformes; Dactylopteroidei; Dactylopteridae; Dactylopterus	*Helicolenus dactylopterus dactylopterus*	(Jamieson, 1991)	2	Canonical	Canonical	External spawning	(Muñoz et al., 2010)
Vertebrata; Gnathostomata; Teleostomi; Euteleostomi; Actinopterygii; Actinopteri; Neopterygii; Teleostei; Osteoglossocephalai; Clupeocephala; Euteleosteomorpha; Neoteleostei; Eurypterygia; Ctenosquamata; Acanthomorphata; Euacanthomorphacea; Percomorphaceae; Syngnathiaria; Syngnathiformes; Mulloidea; Mullidae; Parupeneus	*Paraupeneus spilurus*	(Gwo et al., 2004b)	2	Canonical	Canonical	External - spawning	(Bandai et al., 2020)
Vertebrata; Gnathostomata; Teleostomi; Euteleostomi; Actinopterygii; Actinopteri; Neopterygii; Teleostei; Osteoglossocephalai; Clupeocephala; Euteleosteomorpha; Neoteleostei; Eurypterygia; Ctenosquamata; Acanthomorphata; Euacanthomorphacea; Percomorphaceae; Syngnathiaria; Syngnathiformes; Mulloidea; Mullidae; Parupeneus	*Parupeneus spilurus* (Blackspot goatfish)	(Gwo et al., 2004a)	2	Canonical	Canonical	External spawning	(Bandai et al., 2020)
	Parupeneus multifasciatus	(Emel'yanova and Pavlov, 2012)	2	Canonical	Canonical	External spawning	(Bandai et al., 2020)
	Parupeneus barberinoides	(Emel'yanova and Pavlov, 2012)	2	Canonical	Canonical	External spawning	(Bandai et al., 2020)
Gnathostomata; Teleostomi; Euteleostomi; Actinopterygii; Actinopteri; Neopterygii; Teleostei; Osteoglossocephalai; Clupeocephala; Euteleosteomorpha; Neoteleostei; Eurypterygia; Ctenosquamata; Acanthomorphata; Euacanthomorphacea; Percomorphaceae; Syngnathiaria; Syngnathiformes; Mulloidea; Mullidae; Pseudupeneus	*Pseudupeneus prayensis*	(Jamieson, 1991)	2	Canonical	Canonical	External spawning	(Konoyima and Seisay)
Gnathostomata; Teleostomi; Euteleostomi; Actinopterygii; Actinopteri; Neopterygii; Teleostei; Osteoglossocephalai; Clupeocephala; Euteleosteomorpha; Neoteleostei; Eurypterygia; Ctenosquamata; Acanthomorphata; Euacanthomorphacea; Percomorphaceae; Carangaria; Carangiformes; Carangidae; Trachurus	*Trachurus mediterraneus*	(Jamieson, 1991)	2	Canonical	Canonical	External spawning	(Demirel and Yüksek, 2013)
Gnathostomata; Teleostomi; Euteleostomi; Actinopterygii; Actinopteri; Neopterygii; Teleostei; Osteoglossocephalai; Clupeocephala; Euteleosteomorpha; Neoteleostei; Eurypterygia; Ctenosquamata; Acanthomorphata; Euacanthomorphacea; Percomorphaceae; Carangaria; Carangaria incertae sedis	Galeoides dedactylus	(Jamieson, 1991)	2	Canonical	Canonical	External spawning	(Onyia, 1973)
Gnathostomata;Teleostomi; Euteleostomi; Actinopterygii; Actinopteri; Neopterygii; Teleostei; Osteoglossocephalai; Clupeocephala; Euteleosteomorpha; Neoteleostei; Eurypterygia; Ctenosquamata; Acanthomorphata; Euacanthomorphacea; Percomorphaceae; Carangaria; Carangaria incertae sedis; Centropomidae; Lates	*Lates calcarifer*	(Jamieson, 1991)	2	Canonical	Canonical	External spawning	(Moore, 1982)
Vertebrata; Gnathostomata; Teleostomi; Euteleostomi; Actinopterygii; Actinopteri; Neopterygii; Teleostei; Osteoglossocephalai; Clupeocephala; Euteleosteomorpha; Neoteleostei; Eurypterygia; Ctenosquamata; Acanthomorphata; Euacanthomorphacea; Percomorphaceae; Carangaria; Istiophoriformes; Istiophoridae; Istiompax	*Makaira indica*	(van der Straten et al., 2006)	2	Canonical	Canonical	External- spawning	(de Sylva and Breder, 1997)
Vertebrata; Gnathostomata; Teleostomi; Euteleostomi;	*Kareius bicoloratus*	(Kang et al., 2016)	2	Canonical	Canonical	External - spawning	(Uehara and Shimizu,

Actinopterygii; Actinopteri; Neopterygii; Teleostei; Osteoglossocephalai; Clupeocephala; Euteleosteomorpha; Neoteleostei; Eurypterygia; Ctenosquamata; Acanthomorphata; Euacanthomorphacea; Percomorphaceae; Carangaria; Pleuronectiformes; Pleuronectoidei; Pleuronectidae; Kareius							1996)
Vertebrata; Gnathostomata; Teleostomi; Euteleostomi; Actinopterygii; Actinopteri; Neopterygii; Teleostei; Osteoglossocephalai; Clupeocephala; Euteleosteomorpha; Neoteleostei; Eurypterygia; Ctenosquamata; Acanthomorphata; Euacanthomorphacea; Percomorphaceae; Carangaria; Carangiformes; Carangidae; Seriola	*Seriola dumerilii* (Greater amberjack)	(Maricchiolo et al., 2002)	2	Canonical	Canonical	External spawning	(Marino et al., 1995)
Vertebrata; Gnathostomata; Teleostomi; Euteleostomi; Actinopterygii; Actinopteri; Neopterygii; Teleostei; Osteoglossocephalai; Clupeocephala; Euteleosteomorpha; Neoteleostei; Eurypterygia; Ctenosquamata; Acanthomorphata; Euacanthomorphacea; Percomorphaceae; Carangaria; Pleuronectiformes; Pleuronectoidei; Soleidae; Solea	*Solea senegalensis* (Senegalese sole)	(Medina et al., 2000)	2	Canonical	Canonical	External spawning	(Dinis et al., 1999)
Vertebrata; Gnathostomata; Teleostomi; Euteleostomi; Actinopterygii; Actinopteri; Neopterygii; Teleostei; Osteoglossocephalai; Clupeocephala; Euteleosteomorpha; Neoteleostei; Eurypterygia; Ctenosquamata; Acanthomorphata; Euacanthomorphacea; Percomorphaceae; Carangaria; Carangaria incertae sedis; Centropomidae; Lates	*Nile perch*	(Kahwa et al., 2019)	2	Canonical	Canonical	External spawning	(Kitchell et al., 1997)
Vertebrata; Gnathostomata; Teleostomi; Euteleostomi; Actinopterygii; Actinopteri; Neopterygii; Teleostei; Osteoglossocephalai; Clupeocephala; Euteleosteomorpha; Neoteleostei; Eurypterygia; Ctenosquamata; Acanthomorphata; Euacanthomorphacea; Percomorphaceae; Carangaria; Carangaria incertae sedis; Polynemidae; Polydactylus	*Polydactylus virginicus* (Barbu)	(Gusmao-Pompiani et al., 2005)	2	Canonical	Canonical	External spawning	(Klawe and Alverson, 1964) By *Polydactylus approximans*
Vertebrata; Gnathostomata; Teleostomi; Euteleostomi; Actinopterygii; Actinopteri; Neopterygii; Teleostei; Osteoglossocephalai; Clupeocephala; Euteleosteomorpha; Neoteleostei; Eurypterygia; Ctenosquamata; Acanthomorphata; Euacanthomorphacea; Percomorphaceae; Carangaria; Pleuronectiformes; Pleuronectoidei; Pleuronectidae; Platichthys	*Platichthys tellatus* (Pallas)	(Kwon and Kim, 2014)	2	Canonica	Canonical	External spawning	(Spies and Rice, 1988)
	Platichthys flesus (European flounder)	(Jones and Butler, 1988)	2	Canonical	Canonical	External spawning	(Florin and Höglund, 2008)
Vertebrata; Gnathostomata; Teleostomi; Euteleostomi; Actinopterygii; Actinopteri; Neopterygii; Teleostei; Osteoglossocephalai; Clupeocephala; Euteleosteomorpha; Neoteleostei; Eurypterygia; Ctenosquamata; Acanthomorphata; Euacanthomorphacea; Percomorphaceae; Carangaria; Carangiformes; Rachycentridae; Rachycentron	*Rachycentron canadum* (Cobia)	(Dhanasekar et al., 2018)	2	Canonical	Canonical	External spawning	(Benetti et al., 2008)
Vertebrata; Gnathostomata; Teleostomi; Euteleostomi; Actinopterygii; Actinopteri; Neopterygii; Teleostei; Osteoglossocephalai; Clupeocephala; Otomorpha; Clupei; Clupeiformes; Clupeoidei; Engraulidae; Engraulinae; Engraulis	*Engraulis japonicus*	(Fu et al., 2016)	1 (Confirmed)	Undetected	Canonical	External	(Lee et al., 2006)
Vertebrata; Gnathostomata; Teleostomi; Euteleostomi; Actinopterygii; Actinopteri; Neopterygii; Teleostei; Osteoglossocephalai; Clupeocephala; Otomorpha; Clupei; Clupeiformes; Clupeoidei; Clupeidae; Spratelloidinae; Spratelloides	*Spratelloides gracilis* (Silver-stripe round herring)	(Gwo et al., 2006)	1 (Confirmed)	Undetected	Canonical	External- Spawning- no spermcasting	(Mogi et al., 2009)
Gnathostomata; Teleostomi; Euteleostomi; Actinopterygii; Actinopteri; Neopterygii; Teleostei; Osteoglossocephalai; Clupeocephala; Otomorpha; Clupei; Clupeiformes; Clupeoidei; Clupeidae; Clupeinae; Sardinops	*Sardinops melanostictus*	(Jamieson, 1991)	2	Canonical	Canonical	External spawning	(Watanabe et al., 1996)
Gnathostomata; Teleostomi; Euteleostomi; Actinopterygii; Actinopteri; Neopterygii; Teleostei; Osteoglossocephalai; Clupeocephala; Euteleosteomorpha; Neoteleostei; Eurypterygia; Ctenosquamata; Acanthomorphata; Euacanthomorphacea; Percomorphaceae; Carangaria; Pleuronectiformes; Pleuronectoidei; Paralichthyidae; Paralichthys	*Paralichthys olivaceus*<br>*Paralichthys flesus*	(Jamieson, 1991)	2	Canonical	Canonical	External spawning	(Yasuda et al., 2013)

Gnathostomata; Teleostomi; Euteleostomi; Sarcopterygii; Dipnotetrapodomorpha; Dipnomorpha; Ceratodontae; Ceratodontiformes; Ceratodontoidei; Ceratodontidae	*Neoceratodus forsteril*	(Jamieson, 1991)	2	Canonical	Canonical	External spawning	(Espinoza et al., 2013)
Gnathostomata; Teleostomi; Euteleostomi; Sarcopterygii; Dipnotetrapodomorpha; Dipnomorpha; Ceratodontae; Ceratodontiformes; Lepidosirenoidei; Lepidosirenidae; Lepidosiren	*Lepidosiren paradoxa*	(Jamieson, 1991)	2	Canonical	Canonical	External spawning	(Kerr and Sedgwick, 1900)
Vertebrata;Gnathostomata; Teleostomi; Euteleostomi; Sarcopterygii Dipnotetrapodomorpha; Tetrapoda; Amniota; Sauropsida; Sauria; Lepidosauria; Sphenodontia; Sphenodontidae; Sphenodon	*Sphenodon punctatus* (Tuatara)	(Healy and Jamieson, 1994)	2	Canonical	Canonical	Internal – (artificial) insemination	(Lamar et al., 2021)
Vertebrata; Gnathostomata; Teleostomi; Euteleostomi; Actinopterygii; Actinopteri; Neopterygii; Teleostei; Osteoglossocephalai; Clupeocephala; Euteleosteomorpha; Neoteleostei; Eurypterygia; Ctenosquamata; Acanthomorphata; Euacanthomorphacea; Percomorphaceae; Gobiaria; Gobiiformes; Gobioidei; Gobiidae; Oxudercinae; Boleophthalmus	*Boleophthalmus pectinirostris* (Mudskipper)	(Chung, 2008)	2	Canonical	Canonical	External spawning	(Wanshu et al., 2007)
Gnathostomata; Teleostomi; Euteleostomi; Actinopterygii; Actinopteri; Neopterygii; Teleostei; Osteoglossocephalai; Clupeocephala; Euteleosteomorpha; Neoteleostei; Eurypterygia; Ctenosquamata; Acanthomorphata; Euacanthomorphacea; Percomorphaceae; Gobiaria; Kurtiformes; Apogonoidei	*Apogonidae*	(Jamieson, 1991)	2	Canonical	Canonical	External spawning	(Ishihara and Tachihara, 2011)
Vertebrata; Gnathostomata; Teleostomi; Euteleostomi; Actinopterygii; Actinopteri; Neopterygii; Teleostei; Osteoglossocephalai; Clupeocephala; Euteleosteomorpha; Neoteleostei; Eurypterygia; Ctenosquamata; Acanthomorphata; Euacanthomorphacea; Percomorphaceae; Gobiaria; Gobiiformes; Gobioidei; Gobiidae; Benthophilinae; Neogobiini; Neogobius	*Neogobius melanostomus* (Round goby)	(Allen et al., 2004)	2	Canonical	Canonical	External -spawning	(Meunier et al., 2009)
Gnathostomata; Teleostomi; Euteleostomi; Actinopterygii; Actinopteri; Neopterygii; Teleostei; Osteoglossocephalai; Clupeocephala; Euteleosteomorpha; Neoteleostei; Eurypterygia; Ctenosquamata; Acanthomorphata; Euacanthomorphacea; Percomorphaceae; Gobiaria; Gobiiformes; Eleotroidei; Eleotridae; Eleotrinae; Hypseleotris	*Hypseleotris galii*	(Jamieson, 1991)	2	Canonical	Canonical	External spawning	(Mackay, 1973)
Gnathostomata; Teleostomi; Euteleostomi; Sarcopterygii; Coelacanthimorpha; Coelacanthiformes; Coelacanthidae; Latimeria	*Latimeria chalumnae*	(Jamieson, 1991)	2	Canonical	Canonical	internal	(Balon, 1991)
Gnathostomata; Teleostomi; Euteleostomi; Actinopterygii; Actinopteri; Neopterygii; Teleostei; Osteoglossocephalai; Osteoglossocephala; Osteoglossomorpha; Osteoglossiformes; Pantodontidae; Pantodon	*Pantodon buchholzi*	(Jamieson, 1991)	1 (unconfirmed)	Undetected	Canonical	Internal insemination- no spermcasting	(Dymek and Pecio, 2020)
Gnathostomata; Teleostomi; Euteleostomi; Sarcopterygii; Dipnotetrapodomorpha; Tetrapoda; Amphibia; Batrachia; Caudata; Salamandroidea; Plethodontidae; Plethodontinae	*Paronocheilus* (Cardinal fish)	(Jamieson, 1991)	2	Canonical	Canonical	External spawning	(Lahnsteiner, 2003)
Gnathostomata; Teleostomi; Euteleostomi; Sarcopterygii; Dipnotetrapodomorpha; Dipnomorpha; Ceratodontae; Ceratodontiformes; Lepidosirenoidei; Protopteridae	*Protopterus*	(Jamieson, 1991)	2	Canonical	Canonical	External spawning	(MOSILLE and MMNOYA, 1988) By *Protopterus aethiopicus*
Vertebrata; Actinopterygii; Teleostei ; Osteoglossocephalai; Clupeocephala Clupeocephala; Otomorpha; Ostariophysi; Otophysi; Characiphysae; Siluriformes; Siluroidei; Pimelodidae; Rhamdia	*Rhamdia Quelen*	(Quagio-Grassiotto et all., 2005)	2	Canonical	Canonical	External- spawning	(Pereira et al., 2006)
Vertebrata; Gnathostomata; Teleostomi; Euteleostomi; Actinopterygii; Actinopteri; Neopterygii; Teleostei; Osteoglossocephalai; Clupeocephala; Euteleosteomorpha; Lepidogalaxii; Lepidogalaxiiformes; Lepidogalaxiidae; Lepidogalaxias;	*Lepidogalaxias salamandroides* (Dwarf pencilfish)	(Leung, 1988) (Jamieson, 1991)	1 (Confirmed)	Undetected	Canonical	Internal - no spermcasting	(PUSEY and STEWART, 2008)
Vertebrata; Gnathostomata; Teleostomi; Euteleostomi; Actinopterygii; Actinopteri; Neopterygii; Teleostei; Osteoglossocephalai; Clupeocephala; Euteleosteomorpha;	*Gadus morhua* (Atlantic cod)	(Alavi et al., 2021), (Trippel, 2003)	2	Canonical	Canonical	External spawning	(Fordham and Trippel, 1999)

Neoteleostei; Eurypterygia; Ctenosquamata; Acanthomorphata; Paracanthopterygii; Zeiogadaria; Gadariae; Gadiformes; Gadoidei; Gadidae; Gadus							
Vertebrata; Gnathostomata; Teleostomi; Euteleostomi; Actinopterygii; Actinopteri; Neopterygii; Teleostei; Osteoglossocephalai; Clupeocephala; Euteleosteomorpha; Neoteleostei; Eurypterygia; Ctenosquamata; Acanthomorphata; Euacanthomorphacea; Percomorphaceae; Pelagiaria; Scombriformes; Scombridae; Scombrinae; Thunnini; Thunnus	*Thunnus thynnus* (Bluefin tuna)	(Abascal et al., 2004)	2	Canonica	Canonical	External spawning	(Karakulak et al., 2004)
Vertebrata; Gnathostomata; Teleostomi; Euteleostomi; Actinopterygii; Actinopteri; Neopterygii; Teleostei; Osteoglossocephalai; Clupeocephala; Euteleosteomorpha; Neoteleostei; Eurypterygia; Ctenosquamata; Acanthomorphata; Euacanthomorphacea; Percomorphaceae; Pelagiaria; Scombriformes; Stromateidae; Pampus	*Pampus argenteus*	(Chung et al., 2010)	2	Canonical	Canonical	External spawning	(Almatar et al., 2004)
Gnathostomata; Teleostomi; Euteleostomi; Actinopterygii; Actinopteri; Neopterygii; Teleostei; Osteoglossocephalai; Clupeocephala; Euteleosteomorpha; Neoteleostei; Eurypterygia; Ctenosquamata; Acanthomorphata; Euacanthomorphacea; Percomorphaceae; Pelagiaria; Scombriformes; Scombridae; Scombrinae; Scombrini	*Scomber ustralicus*<br>*Scomber japonicus*<br>*Scomber Tritor*<br>*Thunnus thynnus*<br>*Euthynnus alletteratus*	(Jamieson, 1991)	2	Canonical	Canonical	External spawning	(Dickerson et al., 1992)
Vertebrata; Gnathostomata; Teleostomi; Euteleostomi; Actinopterygii; Actinopteri; Neopterygii; teleostei	*Elopiformes,*<br>*Anguilliformes,*<br>*Notacanthiformes*	(Jamieson and Mattel, 2009)<br>(Parenti and Johnson, 1996)	2	Canonical<br>PC is modified; there is extension of the proximal centriole as two elongate bundles of 4 and 5 triplets	Canonical	External spawning	(Almatar et al., 2004)
Gnathostomata; Teleostomi; Euteleostomi; Actinopterygii; Actinopteri; Neopterygii; Teleostei; Osteoglossocephalai; Clupeocephala; Euteleosteomorpha; Neoteleostei; Eurypterygia; Ctenosquamata; Myctophata; Myctophiformes; Myctophidae; Lampanyctus	*Lampanyctus crocodilus* (Jewel lanternfish)	(Ribes et al., 2015)<br>(Jamieson, 1991)	2	Canonical,<br>Two parallel centrioles that give rise to two flagella of the 9+0 type	Canonical	External spawning- no spermcasting	(Fanelli et al., 2014)
Gnathostomata; Teleostomi; Euteleostomi; Actinopterygii; Actinopteri; Neopterygii; Teleostei; Elopocephalai; Elopocephala	*Elopomorpha*	(Jamieson, 1991)	2	Canonical	Canonical	External spawning	(Arai and Chino, 2018)

References

A. Sabry ., D., and et al. 2021. Comparative histological and functional studies on the brain of some freshwater fishes during prespawning and spawning seasons. *Egyptian Journal of Aquatic Biology and Fisheries*. 25:671-695.

Abascal, F.J., C. Megina, and A. Medina. 2004. Testicular development in migrant and spawning bluefin tuna (Thunnus thynnus (L.)) from the eastern Atlantic and Mediterranean. *Fishery Bulletin*. 102:407-417.

Acha, E.M., H. Mianzan, C.A. Lasta, and R.A. Guerrero. 1999. Estuarine spawning of the whitemouth croaker Micropogonias furnieri (Pisces: Sciaenidae), in the Río de la Plata, Argentina. *Marine and Freshwater Research*. 50:57-65.

Ak, O., S. Kutlu, and I. Karayücel. 2011. Some reproductive characteristics of Uranoscopus scaber Linnaeus, 1758 (Pisces: Uranoscopidae) in the Black Sea (Turkey). *CBM-Cahiers de Biologie Marine*. 52:253.

Alavi, S.M., B. Drozd, A. Hatef, and M. Flajshans. 2013. Sperm morphology, motility, and velocity in naturally occurring polyploid European weatherfish (Misgurnus fossilis L.). *Theriogenology*. 80:153-160.

Alavi, S.M.H., A. Hatef, I.A. Butts, O. Bondarenko, J. Cosson, and I. Babiak. 2021. Some recent data on sperm morphology and motility kinetics in Atlantic cod (Gadus morhua L.). *Fish physiology and biochemistry*. 47:327-338.

Alex Hesp, S., I.C. Potter, and N.G. Hall. 2004. Reproductive Biology and Protandrous Hermaphroditism in Acanthopagrus latus. *Environmental Biology of Fishes*. 70:257-272.

Alhassan, E., S. Abobi, S. Mensah, and F. Boti. 2014. The spawning pattern, length-weight relationship and condition factor of elephant fish, Mormyrus rume from the Bontanga reservoir, Ghana.

Aliniya, M., H. Khara, S.B. Noveiri, and H. Dadras. 2013. Influence of age of common carp (Cyprinus carpio) broodstock on reproductive traits and fertilization. *Turkish Journal of Fisheries and Aquatic Sciences*. 13.

Allen, J.D., G.K. Walker, S.J. Nichols, and D. Sorenson. 2004. The fine structure of the sperm of the round goby (Neogobius melanostomus). *Journal of Great Lakes Research*. 30:566-572.

Almatar, S.M., K.P. Lone, T.S. Abu-Rezq, and A.A. Yousef. 2004. Spawning frequency, fecundity, egg weight and spawning type of silver pomfret, Pampus argenteus (Euphrasen) (Stromateidae), in Kuwait waters. *Journal of Applied Ichthyology*. 20:176-188.

Almeida, E.M.d., and C.L.d.B. Rossi-Wongtschowski. 2007. Maurolicus stehmanni Parin & Kobyliansky, 1993 (Sternoptychidae): length of first maturation, and spawning seasons in the south-southeast Brazilian region. *Brazilian Journal of Oceanography*. 55:309-322.

Arai, T., and N. Chino. 2018. Opportunistic migration and habitat use of the giant mottled eel Anguilla marmorata (Teleostei: Elopomorpha). *Scientific Reports*. 8:5666.

Araujo, R.B.d., and V. Garutti. 2002. Biologia reprodutiva de Apidoras fuscoguttatus (Siluriformes, Callichthyidae) em riacho de cabeceira da bacia do alto Rio Paraná. *Iheringia. Série Zoologia*. 92:89-98.

Asai, T. 1971. Fine structure of centrioral complex in spermiogenesis of the viviparous teleost fish Lebistes reticulatus. *J. Nara med. Ass*. 22:371-382.

Azevedo, M., C. Fialho, and L. Malabarba. 2016. Reproductive strategies in two inseminating species of Glandulocaudini, Mimagoniates microlepis and Mimagoniates rheocharis (Characiformes: Characidae: Stevardiinae). *Journal of Fish Biology*. 89:431-444.

Azevedo, M.A., L.R. Malabarba, and J.R. Burns. 2010. Reproductive biology and development of gill glands in the inseminating characid, Macropsobrycon uruguayanae Eigenmann, 1915 (Cheirodontinae: Compsurini). *Neotropical Ichthyology*. 8:87-96.

Azevedo, M.A., L.R. Malabarba, and C.B. Fialho. 2000. Reproductive biology of the inseminating glandulocaudine Diapoma speculiferum Cope (Teleostei: Characidae). *Copeia*. 2000:983-989.

Badcock, J., P. Whitehead, M. Bauchot, J. Hureau, J. Nielsen, and E. Tortonese. 1984. Fishes of the North-Eastern Atlantic and the Mediterranean. UNESCO, Paris.

Baicere-Silva, C.M., K.M. Ferreira, L.R. Malabarba, R.C. Benine, and I. Quagio-Grassiotto. 2011. Spermatic characteristics and sperm evolution on the subfamily Stevardiinae (Ostariophysi: Characiformes: Characidae). *Neotropical Ichthyology*. 9:377-392.

Balon, E.K. 1991. Probable evolution of the coelacanth's reproductive style: lecithotrophy and orally feeding embryos in cichlid fishes and in Latimeria chalumnae. *Environmental Biology of Fishes*. 32:249-265.

Bandai, A., T. Matsubara, R. Goto, T. Hayakawa, Y. Iwatsuki, and H. Motomura. 2020. Sexual dichromatism and dimorphism in the goatfish Parupeneus spilurus (Perciformes: Mullidae) in southern Japan. *Ichthyological Research*. 67:203-211.

Barcellos, L.J., G.F. Wassermann, A.P. Scott, V.M. Woehl, R.M. Quevedo, I. Ittzés, M.H. Krieger, and F. Lulhier. 2001. Steroid profiles in cultured female jundia, the siluridae Rhamdia quelen (Quoy and Gaimard, Pisces Teleostei), during the first reproductive cycle. *General and Comparative Endocrinology*. 121:325-332.

Barimo, J.F., J.E. Serafy, P.E. Frezza, and P.J. Walsh. 2007. Habitat use, urea production and spawning in the gulf toadfish Opsanus beta. *Marine Biology*. 150:497-508.

Beirão, J., J. Lewis, and C. Purchase. 2015. Spermatozoa ultrastructure of two osmerid fishes in the context of their family (Teleostei: Osmeriformes: Osmeridae). *Journal of Applied Ichthyology*. 31:28-33.

Benetti, D.D., M.R. Orhun, B. Sardenberg, B. O'Hanlon, A. Welch, R. Hoenig, I. Zink, J.A. Rivera, B. Denlinger, D. Bacoat, K. Palmer, and F. Cavalin. 2008. Advances in hatchery and grow-out technology of cobia Rachycentron canadum (Linnaeus). *Aquaculture Research*. 39:701-711.

Bialetzki, A., K. Nakatani, P.V. Sanches, and G. Baumgartner. 2004. Eggs and larvae of the ‘curvina’ Plagioscion squamosissimus (Heckel, 1840) (Osteichthyes, Sciaenidae) in the Baía River, Mato Grosso do Sul State, Brazil. *Journal of Plankton Research*. 26:1327-1336.

Billard, R., A.-M. Escaffre, and N. Tramasaygues. 1970. La spermatogenèse de Poecilia reticulata. IV.—La spermiogenèse. Etude ultrastructurale. *In* Annales de Biologie Animale Biochimie Biophysique. Vol. 10. EDP Sciences. 493-510.

Bohlen, J. 2008. First report on the spawning behaviour of a golden spined loach, Sabanejewia vallachica (Teleostei: Cobitidae). *FOLIA ZOOLOGICA-PRAHA-*. 57:139.

Boschetto, C., C. Gasparini, and A. Pilastro. 2011. Sperm number and velocity affect sperm competition success in the guppy (Poecilia reticulata). *Behavioral Ecology and Sociobiology*. 65:813-821.

Brewer, H.B. 1979. Some preliminary studies of the effects of a static magnetic field on the life cycle of the Lebistes reticulatus (guppy). *Biophysical Journal*. 28:305-314.

Bruslé, S. 1981. Ultrastructure of spermiogenesis in Liza aurata risso, 1810 (Teleostei, Mugilidae). *Cell and tissue research*. 217:415-424.

Burns, J.R., and S.H. Weitzman. 2006. Intromittent organ in the genus Monotocheirodon (Characiformes: Characidae). *Copeia*. 2006:529-534.

Bustos, C.A., F. Balbontín, and M.F. Landaeta. 2007. Spawning of the southern hake Merluccius australis (Pisces: Merlucciidae) in Chilean fjords. *fisheries research*. 83:23-32.

Buzollo, H., R. Veríssimo-Silveira, I.R. Oliveira-Almeida, J.S. Alexandre, H.T. Okuda, and A. Ninhaus-Silveira. 2011. Structural analysis of the Pimelodus maculatus (Lacépède, 1803) embryogenesis (Siluriformes: Pimelodidae). *Neotropical Ichthyology*. 9:601-616.

Cai, Z., Y. Wang, J. Hu, J. Zhang, and Y. Lin. 2010. Reproductive biology of Scatophagus argus and artificial induction of spawning. *J Trop Oceanogr*. 29:180-185.

Camargo, M., and V. Isaac. 2005. Reproductive biology and spatio-temporal distribution of Stellifer rastrifer, Stellifer naso and Macrodon ancylodon (Sciaenidae) in the Caeté estuary, northern Brazil. *Brazilian Journal of Oceanography*. 53:13-21.

Carscadden, J., B.S. Nakashima, and K.T. Frank. 1997. Effects of fish length and temperature on the timing of peak spawning in capelin (Mallotus villosus). *Canadian Journal of Fisheries and Aquatic Sciences*. 54:781-787.

Cassel, M., A. Ferreira, and M. Mehanna. 2014. Ultrastructural features of spermatogenesis in Melanorivulus punctatus (Cyprinodontiformes: Rivulidae). *Micron*. 62:1-6.

Cassel, M., M. Mehanna, L. Mateus, and A. Ferreira. 2013. Gametogenesis and reproductive cycle of Melanorivulus aff. punctatus (Boulenger, 1895)(Cyprinodontiformes, Rivulidae) in Chapada dos Guimarães, Mato Grosso, Brazil. *Neotropical Ichthyology*. 11:179-192.

Chapman, F.A., D.E. Colle, R.W. Rottmann, and J.V. Shireman. 1998. Controlled Spawning of the Neon Tetra. *The Progressive Fish-Culturist*. 60:32-37.

Chondoma, E.C. 1979. Oblique swimming in characoid fishes with special reference to the genus Nannostomus Gunther 1872. University of British Columbia.

Chung, E.-Y. 2008. Ultrastructure of germ cells, the Leydig cells, and Sertoli cells during spermatogenesis in Boleophthalmus pectinirostris (Teleostei, Perciformes, Gobiidae). *Tissue and Cell*. 40:195-205.

Chung, E.-Y., Y.-C. Yang, H.-W. Kang, K.-H. Choi, J.-C. Jun, and K.-Y. Lee. 2010. Ultrastructure of germ cells and the functions of Leydig cells and Sertoli cells associated with spermatogenesis in Pampus argenteus (Teleostei: Perciformes: Stromateidae). *Zoological Studies*. 49:39-50.

Clemens, H.P., and K.E. Sneed. 1957. The spawning behavior of the channel catfish Ictalurus punctatus. US Fish and Wildlife Service.

Cody, R.P. 1993. Spawning and Nest Guarding in a Canary Islands Population of Parablennius parvicornis. *Copeia*. 1993:1151-1154.

Collazos-Lasso, L., and J. Arias-Castellanos. 2009. Stimulating Ancistrus triradiatus final maturation and spawning. *Revista Orinoquia*. 13:14-19.

Costa, E.F.S., J.F. Dias, and H. Murua. 2015. Reproductive strategy and fecundity of the keystone species Paralonchurus brasiliensis (Teleostei, Sciaenidae): an image processing techniques application. *Environmental Biology of Fishes*. 98:2093-2108.

De Lima, Á.C., and C.A.R.M. Araujo-Lima. 2004. The distributions of larval and juvenile fishes in Amazonian rivers of different nutrient status. *Freshwater Biology*. 49:787-800.

De Lima, Á.C., and C.A. Araujo-Lima. 2004. The distributions of larval and juvenile fishes in Amazonian rivers of different nutrient status. *Freshwater Biology*. 49:787-800.

de Oliveira, C.L., J.R. Burns, L.R. Malabarba, and S.H. Weitzman. 2008. Sperm ultrastructure in the inseminating Macropsobrycon uruguayanae (Teleostei: Characidae: Cheirodontinae). *J Morphol*. 269:691-697.

de Sylva, D.P., and P.R. Breder. 1997. Reproduction, gonad histology, and spawning cycles of north Atlantic billfishes (Istiophoridae). *Bulletin of Marine Science*. 60:668-697.

Demirel, N., and A. Yüksek. 2013. Reproductive biology of Trachurus mediterraneus (Carangidae): a detailed study for the Marmara–Black Sea stock. *Journal of the marine biological Association of the United Kingdom*. 93:357-364.

Demirhan, S.A., and K. Seyhan. 2006. Seasonality of reproduction and embryonic growth of spiny dogfish (Squalus acanthias L., 1758) in the eastern Black Sea. *Turkish Journal of Zoology*. 30:433-443.

Dhanasekar, K., N. Selvakumar, and N. Munuswamy. 2018. Ultrastructure of spermatozoa in cobia, Rachycentron canadum (Linnaeus, 1766). *Anim Reprod Sci*. 189:43-50.

Diana, J.S., P. Hanchin, and N. Popoff. 2015. Movement patterns and spawning sites of muskellunge Esox masquinongy in the Antrim chain of lakes, Michigan. *Environmental biology of fishes*. 98:833-844.

Diatta, Y., A. Seck, M. Diop, and O. Guélorget. 2004. Observations on the reproductive biology of the Blackchin guitarfish, Rhinobatos cemiculus E. Geoffroy Saint-Hilaire, 1817 (Chondrichthyes, Rhinobatidae) from the coast of Senegal (Eastern Tropical Atlantic). *Scientia gerundensis*:19-30.

Dickerson, T.L., B. Macewicz, and J. Hunter. 1992. Spawning frequency and batch fecundity of chub mackerel, Scomber japonicus, during 1985. *CalCOFI Rep*. 33:130-140.

DiLauro, M.N., R.A. Walsh, M. Peiffer, and R.M. Bennett. 2001. Sperm-cell ultrastructure of North American sturgeons. IV. The pallid sturgeon (Scaphirhynchus albus Forbes and Richardson, 1905). *Canadian journal of zoology*. 79:802-808.

Dinis, M.T., L. Ribeiro, F. Soares, and C. Sarasquete. 1999. A review on the cultivation potential of Solea senegalensis in Spain and in Portugal. *Aquaculture*. 176:27-38.

Doi, T., S. Aoyama, and I. Kinoshita. 2004. Ontogeny of the mandarinfish Siniperca chuatsi (Perciformes: Sinipercidae) reared in aquarium. *Ichthyological Research*. 51:337.

Duarte, S., F.G. Araújo, A. Sales, and N. Bazzoli. 2007. Morphology of gonads, maturity and spawning season of Loricariichthys spixii (Siluriformes, Loricariidae) in a subtropical reservoir. *Brazilian Archives of Biology and Technology*. 50:1019-1032.

Dymek, A.M., and A. Pecio. 2020. Spermatogenesis in the inseminating African butterflyfish Pantodon buchholzi (Teleostei: Osteoglossiformes: Pantodontidae) with the revision of residual bodies formation. *Journal of Fish Biology*. 97:1491-1506.

Emel'yanova, N., and D. Pavlov. 2012. Gamete ultrastructure in some species of the family Mullidae from the South China Sea. *Journal of Ichthyology*. 52:639-645.

Espinoza, T., S.M. Marshall, and A.J. McDougall. 2013. SPAWNING OF THE ENDANGERED AUSTRALIAN LUNGFISH (NEOCERATODUS FORSTERI) IN A HEAVILY REGULATED RIVER: A PULSE FOR LIFE. *River Research and Applications*. 29:1215-1225.

Fanelli, E., V. Papiol, J.E. Cartes, and O. Rodriguez-Romeu. 2014. Trophic ecology of Lampanyctus crocodilus on north-west Mediterranean Sea slopes in relation to reproductive cycle and environmental variables. *Journal of Fish Biology*. 84:1654-1688.

Faria, A.M., and E.J. Gonçalves. 2010. Ontogeny of swimming behaviour of two temperate clingfishes, Lepadogaster lepadogaster and L. purpurea (Gobiesocidae). *Marine Ecology Progress Series*. 414:237-248.

Faustino, F., R.C. Silva, C.C. Hilbig, L.C. Makino, J.A. Senhorini, A. Ninhaus-Silveira, and L.S.O. Nakaghi. 2015a. Spermatozoon ultrastructure and semen parameters of Brycon vermelha (Characiformes, Characidae). *Animal Reproduction Science*. 157:17-23.

Faustino, F., R.C.d. Silva, C.C. Hilbig, L.C. Makino, J.A. Senhorini, A. Ninhaus-Silveira, and L.S.O. Nakaghi. 2015b. Spermatozoon ultrastructure and semen parameters of Brycon vermelha (Characiformes, Characidae). *Animal reproduction science*. 157:17-23.

Feddern, H.A. 1965. The spawning, growth, and general behavior of the bluehead wrasse, Thalassoma bifasciatum (Pisces: Labridae). *Bulletin of Marine Science*. 15:896-941.

Felicio, G., J. Cordeiro, B. Dutra-Costa, C. Maximino, G. Branco, P. Quirino, and D. de Siqueira-Silva. 2021. Gonadal characterization of the Amazonian fish Serrapinnus kriegi (Characidae: Cheirodontinae). *Brazilian Journal of Biology*. 83.

Figueroa, E., I. Valdebenito, A.B. Zepeda, C.A. Figueroa, K. Dumorné, R.L. Castillo, and J.G. Farias. 2017. Effects of cryopreservation on mitochondria of fish spermatozoa. *Reviews in Aquaculture*. 9:76-87.

Flamio Jr, R., K.A. Chojnacki, A.J. DeLonay, M.J. Dodson, R.M. Gocker, J.A. Jenkins, J. Powell, and E.J. Heist. 2021. Production of haploid gynogens to inform genomic resource development in the paleotetraploid pallid sturgeon (Scaphirhynchus albus). *Aquaculture*. 538:736529.

Fleming, I.A. 1996. Reproductive strategies of Atlantic salmon: ecology and evolution. *Reviews in fish biology and fisheries*. 6:379-416.

Florin, A.B., and J. Höglund. 2008. Population structure of flounder (Platichthys flesus) in the Baltic Sea: differences among demersal and pelagic spawners. *Heredity*. 101:27-38.

Fonseca Ratton, T., N. Bazzoli, and G. Bastos Santos. 2003. Reproductive biology of Apareiodon affinis (Pisces: Parodontidae) in the Furnas Reservoir, Minas Gerais, Brazil. *Journal of Applied Ichthyology*. 19:387-390.

Fordham, B.S.E., and E.A. Trippel. 1999. Feeding behaviour of cod (Gadus morhua) in relation to spawning. *Journal of Applied Ichthyology*. 15:1-9.

França, G., C. Oliveira, and I. Quagio-Grassiotto. 2007. Ultrastructure of spermiogenesis and spermatozoa of Gymnotus cf. anguillaris and Brachyhypopomus cf. pinnicaudatus (Teleostei: Gymnotiformes). *Tissue and cell*. 39:131-139.

Fu, S.-Y., J.-H. Jiang, W.-X. Yang, and J.-Q. Zhu. 2016. A histological study of testis development and ultrastructural features of spermatogenesis in cultured Acrossocheilus fasciatus. *Tissue and Cell*. 48:49-62.

Fujimoto, T., T. Kataoka, S. Sakao, T. Saito, E. Yamaha, and K. Arai. 2006. Developmental stages and germ cell lineage of the loach (Misgurnus anguillicaudatus). *Zoological Science*. 23:977-989.

Fujimoto, Y., Y. Ouchi, T. Hakuba, H. Chiba, and M. Iwata. 2008. Influence of modern irrigation, drainage system and water management on spawning migration of mud loach, Misgurnus anguillicaudatus C. *Environmental Biology of Fishes*. 81:185-194.

Fürböck, S., F. Lahnsteiner, and R.A. Patzner. 2009. A fine structural review on the spermatozoa of Cyprinidae with attention to their phylogenetic implications. *Histology and histopathology*.

Fürböck, S., R.A. Patzner, and F. Lahnsteiner. 2010. Fine structure of spermatozoa of Chondrostoma nasus and Rutilus meidingerii (Teleostei, Cyprinidae), as revealed by scanning and transmission electron microscopy. *Acta Zoologica*. 91:88-95.

Furness, A.I., D.N. Reznick, M.S. Springer, and R.W. Meredith. 2015. Convergent evolution of alternative developmental trajectories associated with diapause in African and South American killifish. *Proceedings of the Royal Society B: Biological Sciences*. 282:20142189.

Gallego, V., L. Pérez, J.F. Asturiano, and M. Yoshida. 2013. Relationship between spermatozoa motility parameters, sperm/egg ratio, and fertilization and hatching rates in pufferfish (Takifugu niphobles). *Aquaculture*. 416-417:238-243.
Gárriz, Á., and L.A. Miranda. 2013. Ultrastructure of fresh and post thawed sperm of pejerrey Odontesthes bonariensis (Atheriniformes). *Neotropical Ichthyology*. 11:831-836.
Gaspare, L., and I. Bryceson. 2013. Reproductive biology and fishery-related characteristics of the Malabar grouper (Epinephelus malabaricus) caught in the coastal waters of Mafia Island, Tanzania. *Journal of Marine Biology*. 2013.
Giora, J., and J.R. Burns. 2011. Sperm ultrastructure in three different families of weakly electric fishes (Teleostei: Gymnotiformes). *Neotropical Ichthyology*. 9:881-888.
Gladstone, W. 1994. Lek-like spawning, parental care and mating periodicity of the triggerfishPseudobalistes flavimarginatus (Balistidae). *Environmental Biology of Fishes*. 39:249-257.
Goff, G.P. 1984. Brood care of longnose gar (Lepisosteus osseus) by smallmouth bass (Micropterus dolomieui). *Copeia*:149-152.
Gonçalves, T.K., M.A. Azevedo, L.R. Malabarba, and C.B. Fialho. 2005. Reproductive biology and development of sexually dimorphic structures in Aphyocharax anisitsi (Ostariophysi: Characidae). *Neotropical Ichthyology*. 3:433-438.
Gordon, C.D. 1965. Aspects of the life-history of Cymatogaster aggregata Gibbons. University of British Columbia.
Goto, A. 1988. Reproductive behavior and homing after downstream spawning migration in the river sculpin, Cottus hangiongensis. *Ichthyological Research*. 34:488-496.
Gratani, M., F. Royee, and J.R.A. Butler. 2016. A research process and criteria–indicators framework for developing indigenous freshwater ecosystem health monitoring. *Cogent Environmental Science*. 2:1214228.
Greven, H., and G. Schmahl. 2006. A note on the spermatozoon ultrastructure of the foureyed fish Anableps anableps (Atherinomorpha, Cyprinodontiformes). *Z. Fischkunde*:83-88.
Grier, H. 1976. Sperm development in the teleost Oryzias latipes. *Cell and Tissue Research*. 168:419-431.
Grier, H.J. 1973. Ultrastructure of the testis in the teleost Poecilia latipinna. Spermiogenesis with reference to the intercentriolar lamellated body. *J Ultrastruct Res*. 45:82-92.
Grönberg, R., and A. Telkkä. 1967. Juxtanuclear changes during the early spermatogenesis in Lebistes reticulatus (Guppy). *Zeitschrift für Zellforschung und Mikroskopische Anatomie*. 84:342-349.
Gronberg, R., and J. Wartiovaara. 1972. FREEZE-ETCHING STUDY OF SPERMIOGENESIS IN A FISH (LEBISTES-RETICULATUS). *In* JOURNAL OF ULTRASTRUCTURE RESEARCH. Vol. 38. ACADEMIC PRESS INC ELSEVIER SCIENCE 525 B ST, STE 1900, SAN DIEGO, CA 92101 …. 197-+.
Guo, W., J. Shao, P. Li, J. Wu, and Q. Wei. 2016. Morphology and ultrastructure of Brachymystax lenok tsinlingensis spermatozoa by scanning and transmission electron microscopy. *Tissue Cell*. 48:321-327.
Gusmao-Pompiani, P., C. Oliveira, and I. Quagio-Grassiotto. 2005. Spermatozoa ultrastructure in Sciaenidae and Polynemidae (Teleostei:Perciformes) with some consideration on Percoidei spermatozoa ultrastructure. *Tissue Cell*. 37:177-191.
GWO, J., H. GWO, Y. KAO, B. LIN, and H. SHIH. 1994. Spermatozoan ultrastructure of two species of grouper Bpinephelus malabaricus and Plectropomus leopardus.
Gwo, J.-C. 1995. Spermatozoan ultrastructure of the teleost fish Acanthopagrus latus (Perciformes: Sparidae) with special reference to the basal body. *Journal of submicroscopic cytology and pathology*. 27:391-391.
Gwo, J.-C., J.-Y. Chiu, C.-Y. Lin, Y. Su, and S.-L. Yu. 2005. Spermatozoal ultrastructure of four Sparidae fishes: Acanthopagrus berda, Acanthopagrus australis, Lagodon rhomboids and Archosargus probatocephus. *Tissue and Cell*. 37:109-115.
Gwo, J.-C., M.-C. Kuo, J.-Y. Chiu, and H.-Y. Cheng. 2004a. Ultrastructure of Pagrus major and Rhabdosargus sarba spermatozoa (Perciformes: Sparidae: Sparinae). *Tissue and Cell*. 36:141-147.
Gwo, J.-C., X. Lin, H. Gwo, H. Wu, and P. Lin. 1996. The ultrastructure of Formosan landlocked salmon, Oncorbynchus masout formosanus, spermatozoon (Teleostei, Salmoniformes, Salmonidae). *Journal of submicroscopic cytology and pathology*. 28:33-40.

Gwo, J.-C., H. Ohta, K. Okuzawa, and H.-C. Wu. 1999a. Cryopreservation of sperm from the endangered Formosan landlocked salmon (Oncorhynchus masou formosanus). *Theriogenology*. 51:569-582.
Gwo, J.C., C.Y. Lin, W.L. Yang, and Y.C. Chou. 2006. Ultrastructure of the sperm of blue sprat, Spratelloides gracilis; Teleostei, Clupeiformes, Clupeidae. *Tissue and Cell*. 38:285-291.
Gwo, J.C., H. Ohta, K. Okuzawa, and H.C. Wu. 1999b. Cryopreservation of sperm from the endangered formosan landlocked salmon (Oncorhynchus masou formosanus). *Theriogenology*. 51:569-582.
Gwo, J.C., W.T. Yang, M.C. Kuo, A. Takemura, and H.Y. Cheng. 2004b. Spermatozoal ultrastructures of two marine perciform teleost fishes, the goatfish, Paraupeneus spilurus (Mullidae) and the rabbitfish, Siganus fuscescens (Siganidae) from Taiwan. *Tissue Cell*. 36:63-69.
Hamed, O., and N. Chakroun-Marzouk. 2017. Aspects of the reproductive biology of Trachinus radiatus Cuvier, 1829 (Pisces: Trachinidae) in the Gulf of Tunis. *Cahiers de Biologie Marine*. 58:435-441.
Hara, S., M.N. Duray, M. Parazo, and Y. Taki. 1986. Year-round spawning and seed production of the rabbitfish, Siganus guttatus. *Aquaculture*. 59:259-272.
Harding, S.M., and M.E. Chittenden. 1987. Reproduction, movements, and population dynamics of the southern kingfish, Menticirrhus americanus, in the northwestern Gulf of Mexico. US Department of Commerce, National Oceanic and Atmospheric Administration ….
Hatef, A., S.M. Alavi, S.B. Noveiri, H. Poorbagher, A.R. Alipour, M. Pourkazemi, and O. Linhart. 2011. Morphology and fine structure of Acipenser persicus (Acipenseridae, Chondrostei) spermatozoon: Inter-species comparison in Acipenseriformes. *Anim Reprod Sci*. 123:81-88.
Hatef, A., S.M.H. Alavi, M. Golshan, and O. Linhart. 2013. Toxicity of environmental contaminants to fish spermatozoa function in vitro—a review. *Aquatic toxicology*. 140:134-144.
Healey, M., P. Kline, and T. Chu-Fa. 2001. Saving the Endangered Formosa Landlocked Salmon. *Fisheries*. 26:6-14.
Healy, J., and B. Jamieson. 1994. The ultrastructure of spermatogenesis and epididymal spermatozoa of the tuatara Sphenodon punctatus (Sphenodontida, Amniota). *Philosophical Transactions of the Royal Society of London. Series B: Biological Sciences*. 344:187-199.
Herjayanto, M., O. Carman, and D.T. Soelistyowati. 2016. Tingkah laku memijah, potensi reproduksi ikan betina, dan optimasi teknik pemijahan ikan pelangi Iriatherina werneri Meinken, 1974 [Spawning behavior, female reproductive potential and breeding technique optimize of threadfin rainbowfish. *Jurnal Iktiologi Indonesia*. 16:171-183.
Hontela, A., and N. Stacey. 2019. Cyprinidae. *In* Reproductive seasonality in teleosts. CRC press. 53-78.
Idahor, K. 2014. Microscopic observation of spermatozoa in milt collected with syringe without sacrificing the male African Catfish (Clarias anguillaris B. 1911). *International Journal of Fisheries and Aquatic Studies*. 2:88-91.
Ishihara, T., and K. Tachihara. 2011. Pelagic Larval Duration and Settlement Size of Apogonidae, Labridae, Scaridae, and Tripterygiidae Species in a Coral Lagoon of Okinawa Island, Southern Japan1. *Pacific Science*. 65:87-93.
Jamieson, B.G. 1989. Complex spermatozoon of the live-bearing half-beak, Hemirhamphodon pogonognathus (Bleeker): ultrastructural description (Euteleostei, Atherinomorpha, Beloniformes). *Gamete Res*. 24:247-259.
Jamieson, B.G. 1991. Fish evolution and systematics: evidence from spermatozoa: with a survey of lophophorate, echinoderm and protochordate sperm and an account of gamete cryopreservation. Cambridge University Press.
Jamieson, B.G., and L.-P. Leung. 1991. Fish evolution and systematics: evidence from spermatozoa: with a survey of lophophorate, echinoderm and protochordate sperm and an account of gamete cryopreservation. Cambridge University Press.
Jamieson, B.G., and X. Mattel. 2009. cchapter 10. *Reproductive Biology and Phylogeny of Fishes (Agnathans and Bony Fishes): Phylogeny, Reproductive System, Viviparity, Spermatozoa*:255.
Jamieson, B.G.M., and H.J. Grier. 1993. Influences of phylogenetic position and fertilization biology on spermatozoal ultrastructure exemplified by exocoetoid and poeciliid fish. *Hydrobiologia*. 271:11-25.
Javonillo, R., J.R. Burns, and S.H. Weitzman. 2007. Reproductive morphology of Brittanichthys axelrodi (Teleostei: Characidae), a miniature inseminating fish from South America. *Journal of Morphology*. 268:23-32.

Johannessen, T., J. Gjøsæter, and E. Moksness. 1993. Reproduction, spawning behaviour and captive breeding of the common wolffish Anarhichas lupus L. *Aquaculture*. 115:41-51.

Jones, P.R., and R.D. Butler. 1988. Spermatozoon ultrastructure of Platichthys flesus. *J Ultrastruct Mol Struct Res*. 98:71-82.

Kahwa, D., E. Nyatia, J. Rutaisire, and H. Kaiser. 2019. Spermatozoa morphology and ultrastructure in Nile perch, Lates niloticus (Linnaeus, 1758). *African Journal of Aquatic Science*. 44:97-102.

Kang, H.-W., S.H. Kim, and J.S. Chung. 2016. Ultrastructural studies of germ cell development and the functions of leydig cells and sertoli cells associated with spermatogenesis in Kareius bicoloratus (Teleostei, Pleuronectiformes, Pleuronectidae). *Development & reproduction*. 20:11.

Karakulak, S., I. Oray, A. Corriero, M. Deflorio, N. Santamaria, S. Desantis, and G. De Metrio. 2004. Evidence of a spawning area for the bluefin tuna (Thunnus thynnus L.) in the eastern Mediterranean. *Journal of Applied Ichthyology*. 20:318-320.

Kawamura, K., and K. Uehara. 2005. Effects of temperature on free-embryonic diapause in the autumn-spawning bitterling Acheilognathus rhombeus(Teleostei: Cyprinidae). *Journal of Fish Biology*. 67:684-695.

Kerr, S.G., and A. Sedgwick. 1900. V. The the external features the development of lepidosiren paradoxa, Fitz. *Philosophical Transactions of the Royal Society of London. Series B, Containing Papers of a Biological Character*. 192:299-330.

Kesiktaş, M., E. Yemişken, T. Yildiz, and L. Eryilmaz. 2020. Age, growth and reproduction of the golden grey mullet, Chelon auratus (Risso, 1810) in the Golden Horn Estuary, Istanbul. *Journal of the Marine Biological Association of the United Kingdom*. 100:989-995.

Khatun, D., M.Y. Hossain, F. Nawer, A.A. Mostafa, and A.A. Al-Askar. 2019. Reproduction of Eutropiichthys vacha (Schilbeidae) in the Ganges River (NW Bangladesh) with special reference to potential influence of climate variability. *Environmental Science and Pollution Research*. 26:10800-10815.

Ki, S.-U., and W.-K. Lee. 2018. The annual reproductive cycle of Silurus microdorsalis, a Korean endemic species. *Development & reproduction*. 22:1.

Kim, K.-H. 2019. Anatomical ultrastructure of spermatozoa of Korean sharpbelly, Hemiculter eigenmanni (Cypriniformes, Cyprinidae). *Korean Journal of Ichthyology*. 31:1-6.

Kim, S.H., C.H. Lee, Y.B. Song, S.W. Hur, H.B. Kim, and Y.D. Lee. 2013. Ultrastructure of late spermatids and spermatozoa during spermiogenesis in longtooth grouper Epinephelus bruneus from Jeju, Korea. *Tissue Cell*. 45:261-268.

KIM, Y.H., Y.J. KANG, and D.K. RYU. 1999. Growth of Ammodytes personatus in Korean waters 1, Daily Growth Increment, Early Growth and Spawning Time in Juvenile Stage. *Korean Journal of Fisheries and Aquatic Sciences*. 32:550-555.

Kirschbaum, F., and C. Schugardt. 2002. Reproductive strategies and developmental aspects in mormyrid and gymnotiform fishes. *Journal of Physiology-Paris*. 96:557-566.

Kitchell, J.F., D.E. Schindler, R. Ogutu-Ohwayo, and P.N. Reinthal. 1997. THE NILE PERCH IN LAKE VICTORIA: INTERACTIONS BETWEEN PREDATION AND FISHERIES. *Ecological Applications*. 7:653-664.

Klawe, W., and F. Alverson. 1964. Occurrence of two species of young threadfin, Polydactylus opercularis and P. approximans, in the offshore waters of the eastern tropical Pacific Ocean.

Knight, J.T., G.L. Butler, P.S. Smith, and R.N.E. Wager. 2007. Reproductive biology of the endangered Oxleyan pygmy perch Nannoperca oxleyana Whitley. *Journal of Fish Biology*. 71:1494-1511.

Koch, J., M. Quist, K. Hansen, and G. Jones. 2009. Population dynamics and potential management of bowfin (Amia calva) in the upper Mississippi River. *Journal of Applied Ichthyology*. 25:545-550.

Koger, C.S., S.J. Teh, and D.E. Hinton. 1999. Variations of Light and Temperature Regimes and Resulting Effects on Reproductive Parameters in Medaka (Oryzias latipes)1. *Biology of Reproduction*. 61:1287-1293.

Konoyima, K.J., and L.D. Seisay. Aspects of Reproductive Biology of Pseudupeneus prayensis collected from the coast off Sierra Leone, West Africa. *Journal of Applied Biosciences*. 158:16371-16381.

Kornienko, E.S. 2001. The Spawning Behavior of the Pipefish Syngnathus acusimilis. *Russian Journal of Marine Biology*. 27:54-57.

Kostomarova, A. 1991. The loach Misgurnus fossilis. *In* Animal species for developmental studies. Springer. 125-144.
Kowalski, R., P. Hliwa, A. Andronowska, J. Król, G. Dietrich, M. Wojtczak, R. Stabiński, and A. Ciereszko. 2006. Semen biology and stimulation of milt production in the European smelt (Osmerus eperlanus L.). *Aquaculture*. 261:760-770.
Křišťan, J., A. Hatef, S. Alavi, and T. Policar. 2014. Sperm morphology, ultrastructure, and motility in pikeperch Sander lucioperca (Percidae, Teleostei) associated with various activation media. *Czech J Anim Sci*. 59:1-10.
Kroll, W. 1984. Morphological and behavioral embryology and spontaneous diapause in the African killifish, Aphyosemion gardneri. *Environmental Biology of Fishes*. 11:21-28.
Kudo, S., O. Linhart, and R. Billard. 1994. Ultrastructural studies of sperm penetration in the egg of the European catfish, Silurus glanis. *Aquatic Living Resources*. 7:93-98.
Kwon, A.S., and K.H. Kim. 2014. Anatomical Ultrastructure of Spermatozoa of Platichthys stellatus (Pleuronectidae, Pleuronectiformes) from Korea. *Korean Journal of Ichthyology*. 26:274-280.
Lahnsteiner, F. 2003. The spermatozoa and eggs of the cardinal fish. *Journal of Fish Biology*. 62:115-128.
Lahnsteiner, F., and N. Mansour. 2004. Sperm fine structure of the pikeperch, Sander lucioperca (Percidae, Teleostei). *J Submicrosc Cytol Pathol*. 36:309-312.
Lamar, S.K., N.J. Nelson, J.A. Moore, H.R. Taylor, S.N. Keall, and D.K. Ormsby. 2021. Initial collection, characterization, and storage of tuatara (Sphenodon punctatus) sperm offers insight into their unique reproductive system. *Plos one*. 16:e0253628.
Lappalainen, J., H. Dörner, and K. Wysujack. 2003. Reproduction biology of pikeperch (Sander lucioperca (L.)) – a review. *Ecology of Freshwater Fish*. 12:95-106.
Lee, C.-S., C. Tamaru, C. Kelley, and J. Banno. 1986. Induced spawning of milkfish, Chanos chanos, by a single application of LHRH-analogue. *Aquaculture*. 58:87-98.
Lee, H.-W., J.-H. Kim, and Y.-J. Kang. 2006. Sexual maturation and spawning in the sandfish Arctoscopus japonicus in the east sea of Korea. *Korean Journal of Fisheries and Aquatic Sciences*. 39:349-356.
Lee, Y.H., and K.H. Kim. 2001. The ultrastructure of spermatozoa of the slender catfish Silurus microdorsalis (Teleostei, Siluriformes, Siluridae) with phylogenetic considerations. *J Submicrosc Cytol Pathol*. 33:329-336.
Leung, L.K. 1988. Ultrastructure of the spermatozoon of Lepidogalaxias salamandroides and its phylogenetic significance. *Gamete Res*. 19:41-49.
Lim, H.K., and M.H. Le. 2013. Evaluation of extenders and cryoprotectants on motility and morphology of longtooth grouper (Epinephelus bruneus) sperm. *Theriogenology*. 79:867-871.
Linhart, O., M. Rodina, M. Kocour, and D. Gela. 2006. Insemination, fertilization and gamete management in tench, Tinca tinca (L.). *Aquaculture International*. 14:61-73.
Little, D., D. Macintosh, and P. Edwards. 1993a. Improving spawning synchrony in the Nile tilapia, Oreochromis niloticus (L.). *Aquaculture Research*. 24:399-405.
LITTLE, D.C., D.J. MACINTOSH, and P. EDWARDS. 1993b. Improving spawning synchrony in the Nile tilapia, Oreochromis niloticus (L.). *Aquaculture Research*. 24:399-405.
Lo Nostro, F., H. Grier, L. Andreone, and G. Guerrero. 2003. Involvement of the gonadal germinal epithelium during sex reversal and seasonal testicular cycling in the protogynous swamp eel, Synbranchus marmoratus Bloch 1795 (Teleostei, Synbranchidae). *Journal of Morphology*. 257:107-126.
Luo, D., J.J. Sun, X. Lu, L.Z. Liu, S.J. Chen, and G.F. Li. 2011. Comparative sperm ultrastructure of three species in Siniperca (Teleostei: Perciformes: Sinipercidae). *Micron*. 42:884-891.
Lyle, A.A., and P.S. Maitland. 1997. The spawning migration and conservation of smelt Osmerus eperlanus in the River Cree, southwest Scotland. *Biological Conservation*. 80:303-311.
Mackay, N. 1973. The reproductive cycle of the firetail gudgeon, Hypseleotris galii I. Seasonal histological changes in the ovary. *Australian Journal of Zoology*. 21:53-66.

Madhavi, M., M. Kailasam, and D.L. Mohanlal. 2015. Ultrastructure of sperm of the Spotted scat (Scatophagus argus, Linnaeus, 1766) observed by scanning and transmission electron microscopy. *Anim Reprod Sci*. 153:69-75.

Malmqvist, B. 1980. The spawning migration of the brook lamprey, Lampetra planeri Bloch, in a South Swedish stream. *Journal of Fish Biology*. 16:105-114.

Mann, D.A., J. Bowers-Altman, and R.A. Rountree. 1997. Sounds produced by the striped cusk-eel Ophidion marginatum (Ophidiidae) during courtship and spawning. *Copeia*. 1997:610-612.

Mariac, C., J.F. Renno, Y. Vigouroux, E. Mejia, C. Angulo, D. Castro Ruiz, G. Estivals, C. Nolorbe, A. García Vasquez, and J. Nuñez. 2021. Species-level ichthyoplankton dynamics for 97 fishes in two major river basins of the Amazon using quantitative metabarcoding. *Molecular Ecology*.

Maricchiolo, G., L. Genovese, R. Laura, V. Micale, and U. Muglia. 2002. The ultrastructure of amberjack (Seriola dumerilii) sperm. *Eur J Morphol*. 40:289-292.

Maricchiolo, G., R. Laura, L. Genovese, M.C. Guerrera, V. Micale, and U. Muglia. 2010. Fine structure of spermatozoa in the blackspot sea bream Pagellus bogaraveo (Brunnich, 1768) with some considerations about the centriolar complex. *Tissue Cell*. 42:88-96.

Marino, G., A. Mandich, A. Massari, F. Andaloro, S. Porrello, M.G. Finoia, and F. Cevasco. 1995. Aspects of reproductive biology of the Mediterranean amberjack (Seriola dumerilii Risso) during the spawning period. *Journal of Applied Ichthyology*. 11:9-24.

Markova, M.D., and R.S. Zhivkova. 2003a. Possible cytoskeletal structures of rainbow trout sperm revealed by electron microscopic observation after detergent extraction. *Animal reproduction science*. 79:127-132.

Markova, M.D., and R.S. Zhivkova. 2003b. Possible cytoskeletal structures of rainbow trout sperm revealed by electron microscopic observation after detergent extraction. *Anim Reprod Sci*. 79:127-132.

Marraro, F., M. de los Angeles Bistoni, and M. Carranza. 2005. Spawning season, ovarian development and fecundity of female Trichomycterus corduvense (Osteichthyes, Siluriformes). *Hydrobiologia*. 534:223-230.

Martins, Y.S., F.P. Arantes, Y. Sato, J.E. dos Santos, E. Rizzo, and N. Bazzoli. 2012. Comparative analysis of gonadal morphology in six fish species of the Incertae Sedis genera in Characidae of occurrence in the São Francisco River Basin, Brazil. *Acta Zoologica*. 93:48-56.

Matos, E., M.N. Santos, and C. Azevedo. 2002. Biflagellate spermatozoon structure of the hermaphrodite fish Satanoperca jurupari (Heckel, 1840) (Teleostei, Cichlidae) from the Amazon River. *Braz J Biol*. 62:847-852.

Matsumura, K., S. Matsunaga, and N. Fusetani. 2004. Possible involvement of phosphatidylcholine in school recognition in the catfish, Plotosus lineatus. *Zoological science*. 21:257-264.

Matsuyama, M., S. Adachi, Y. Nagahama, and S. Matsuura. 1988. Diurnal rhythm of oocyte development and plasma steroid hormone levels in the female red sea bream, Pagrus major, during the spawning season. *Aquaculture*. 73:357-372.

Mattei, X. 1970. Spermiogenèse comparée des poissons. *Academic Press*:57-69

.

Mattei, X., and C. Boisson. 1966. Le complexe centriolaire du spermatozoïde de Lebistes reticulatus. *Comptes Rendus Hebdomadaires des Seances de l Academie des Sciences Serie D*. 262:2620-&.

Maulana, F., D.T. Soelistyowati, and M.F. Furqon. 2021. SPAWNING OF BLACK GHOST KNIFEFISH, Apteronotus albifrons WITH DIFFERENT SEX RATIOS. *Indonesian Aquaculture Journal*. 16:29-34.

McEachran, J., and J.D. Fechhelm. 2021. Semionotiformes. *In* Fishes of the Gulf of Mexico, Vol. 1: Myxiniformes to Gasterosteiformes. University of Texas Press. 199-203.

Medina, A., C. Megina, F.J. Abascal, and A. Calzada. 2000. The spermatozoon morphology of Solea senegalensis (Kaup, 1858) (Teleostei, Pleuronectiformes). *J Submicrosc Cytol Pathol*. 32:645-650.

Meinelt, B.T., C. Schulz, M. Wirth, H. Kürzinger, and C. Steinberg. 1999. Dietary fatty acid composition influences the fertilization rate of zebrafish(Danio rerio Hamilton-Buchanan). *Journal of Applied Ichthyology*. 15:19-23.

Meneguelli De Souza, L.C., C.A. Retamal, G.M. Rocha, and M.L. Lopez. 2015. Morphological evidence for a permeability barrier in the testis and spermatic duct of Gymnotus carapo (Teleostei: Gymnotidae). *Molecular Reproduction and Development*. 82:663-678.

Menezes, N.A., S.H. Weitzman, and I. Quagio-Grassiotto. 2013. Two new species and a review of the inseminating freshwater fish genus Monotocheirodon (Characiformes: Characidae) from Peru and Bolivia. *Papéis Avulsos de Zoologia*. 53:129-144.

Meunier, B., S. Yavno, S. Ahmed, and L.D. Corkum. 2009. First documentation of spawning and nest guarding in the laboratory by the invasive fish, the round goby (Neogobius melanostomus). *Journal of Great Lakes Research*. 35:608-612.

Miao, L., M. Li, X. Tang, T. Wang, X. Zhang, and T. Wang. 2013. Sperm Ultrastructure of Miichthys miiuy (Perciformes: Sciaenidae). *The Israeli Journal of Aqua-culture*. 64:1-7.

Micale, V., G. Maricchiolo, and L. Genovese. 2002. The reproductive biology of blackspot sea bream Pagellus bogaraveo in captivity. I. gonadal development, maturation and hermaphroditism. *Journal of Applied Ichthyology*. 18:172-176.

Militelli, M.I., and G.J. Macchi. 2006. Spawning and fecundity of striped weakfish, Cynoscion guatucupa, in the Río de la Plata estuary and adjacent marine waters, Argentina–Uruguay. *Fisheries Research*. 77:110-114.

MILTON, D.A., and A.H. ARTHINGTON. 1983. Reproduction and growth of Craterocephalus marjoriae and C. stercusmuscarum (Pisces: Atherinidae) in south-eastern Queensland, Australia. *Freshwater Biology*. 13:589-597.

Miranda, L., G. Berasain, C. Velasco, Y. Shirojo, and G. Somoza. 2006. Natural spawning and intensive culture of pejerrey Odontesthes bonariensis juveniles. *Biocell*. 30:157-162.

Miranda, M., A.C. Silva, and P.K. Stoddard. 2008. Use of space as an indicator of social behavior and breeding systems in the gymnotiform electric fish Brachyhypopomus pinnicaudatus. *Environmental Biology of Fishes*. 83:379-389.

Mogi, K., K. Misawa, K. Utsunomiya, Y. Kawada, T. Yamazaki, S. Takeuchi, and R. Toyoizumi. 2009. Optic chiasm in the species of order Clupeiformes, family Clupeidae: Optic chiasm of Spratelloides gracilis shows an opposite laterality to that of Etrumeus teres. *Laterality*. 14:495-514.

Moore, R. 1982. Spawning and early life history of burramundi, <I>Lates calcarifer</I> (Bloch), in Papua New Guinea. *Marine and Freshwater Research*. 33:647-661.

Morisawa, S. 2001. Ultrastructural studies of late-stage spermatids and mature spermatozoa of the puffer fish, Takifugu niphobles (Tetraodontiformes) and the effects of osmolality on spermatozoan structure. *Tissue and Cell*. 33:78-85.

Morisawa, S. 2005. Spermiogenesis in the Hagfish Eptatretus burgeri (Agnatha). *Biol Bull*. 209:204-214.

MOSILLE, O.I.I.W., and J.R. MMNOYA. 1988. Reproductive biology of the East African lungfish (Protopterus aethiopicus) in Mwanza Gulf, Lake Victoria. *African Journal of Ecology*. 26:149-162.

Munehara, H. 1988. Spawning and subsequent copulating behavior of the elkhorn sculpin Alcichthys alcicornis in an aquarium. *Japanese Journal of Ichthyology*. 35:358-364.

Munehara, H., Z. Kanamoto, and T. Miura. 2000. Spawning behavior and interspecific breeding in three japanese greenlings (hexagrammidae). *Ichthyological Research*. 47:287-292.

Muñoz, M., C. Dimitriadis, M. Casadevall, S. Vila, E. Delgado, J. Lloret, and F. Saborido-Rey. 2010. Female reproductive biology of the bluemouth Helicolenus dactylopterus dactylopterus: spawning and fecundity. *Journal of Fish Biology*. 77:2423-2442.

Nadezhdina, E.S., L.A. Zinovkina, D. Fais, and S. Chentsov Iu. 2001. [Spermatozoa of the loach Misgurnus fossilis as a test system for identification of new centromere proteins]. *Ontogenez*. 32:41-50.

Natal'ya, G., and D.A. Pavlov. 2017. Spermatozoon ultrastructure in three fish species of the suborder Scorpaenoidei. *Life-Supporting Asia-Pacific Marine Ecosystems, Biodiversity and Their Functioning*:148.

Needham, R.G. 1965. Spawning of paddlefish induced by means of pituitary material. *The Progressive Fish-Culturist*. 27:13-19.

Neves, A., V. Sequeira, R.B. Paiva, A.R. Vieira, and L.S. Gordo. 2014. Reproductive patterns of blacktail comber (Serranus atricauda, Serranidae) from south-west Portugal seamounts. *Helgoland Marine Research*. 68:133-142.

Niksirat, H., L. Andersson, A. Golpour, L. Chupani, and P. James. 2017. Quantification of egg proteome changes during fertilization in sterlet Acipenser ruthenus. *Biochemical and biophysical research communications*. 490:189-193.

O'Connor, W., and J. Koehn. 1991. Spawning of the mountain galaxias Galaxias olidus Gunther. *Bruces Creek, Victoria. Proceedings of the Royal Society of Victoria*. 103:113-123.

Ohta, T. 1991. Initial stages of sperm-egg fusion in the freshwater teleost, Rhodeus ocellatus ocellatus. *Anat Rec*. 229:195-202.

Ohta, T., K.H. Kato, T. Abe, and T. Takeuchi. 1993. Sperm morphology and distribution of intramembranous particles in the sperm heads of selected freshwater teleosts. *Tissue Cell*. 25:725-735.

olidus is widespread throughout Victoria, G. SPAwNING OF THE MOUNTAIN GALAXIAS, GALAXIAS OLIDUS GÜNTHER.

Oliveira, C.L., C.B. Fialho, and L.R. Malabarba. 2010. Reproductive period, fecundity and histology of gonads of two cheirodontines (Ostariophysi: Characidae) with different reproductive strategies-insemination and external fertilization. *Neotropical Ichthyology*. 8:351-360.

Onyia, A. 1973. A contribution to the food and feeding habit of the threadfin Galeoides decadactylus. *Marine Biology*. 22:371-378.

Otake, T., C. Yamada, and K. Uchida. 2002. Contribution of stocked ayu (Plecoglossus altivelis altivelis) to reproduction in the Nagara River, Japan. *Fisheries science*. 68:948-950.

Oulton, L.J., V. Haviland, and C. Brown. 2013. Predator recognition in rainbowfish, Melanotaenia duboulayi, embryos. *PLoS One*. 8:e76061.

Pankhurst, N., and D. Kime. 1991. Plasma sex steroid concentrations in male blue cod, <I>Parapercis colias</I> (Bloch & Schneider) (Pinguipedidae), sampled underwater during the spawning season. *Marine and Freshwater Research*. 42:129-137.

Parenti, L.R., H.J. Grier, and M. Uribe. 2015. Reproductive biology of Chlorophthalmus agassizi Bonaparte, 1840 (Teleostei: Aulopiformes: Chlorophthalmidae) as revealed through histology of archival museum specimens. *Copeia*. 103:821-837.

Parenti, L.R., and G.D. Johnson. 1996. Intercelationshipt of Fishes.

Parreira, G.G., H. Chiarini-Garcia, R.C. Melo, F.O. Vieira, and H.P. Godinho. 2009a. Spermatozoon and its relationship with the ovarian lamellae in the internally inseminating catfish Trachelyopterus galeatus. *Microsc Res Tech*. 72:889-897.

Parreira, G.G., H. Chiarini-Garcia, R.C.N. Melo, F.O. Vieira, and H.P. Godinho. 2009b. Spermatozoon and its relationship with the ovarian lamellae in the internally inseminating catfish Trachelyopterus galeatus. *Microscopy Research and Technique*. 72:889-897.

Pavlov, D., and N. Emel'yanova. 2018. Comparative analysis of spermatozoa morphology in three fish species from the suborder Scorpaenoidei. *Journal of Ichthyology*. 58:226-238.

Pecio, A. 2003. Spermiogenesis and fine structure of the spermatozoon in a headstander, Chilodus punctatus (Teleostei, Characiformes, Anostomidae). *Folia Biol (Krakow)*. 51:55-62.

Pecio, A. 2008. Ultrastructural examination of spermiogenesis and spermatozoon ultrastructure in Congo tetra Phenacogrammus interruptus Boulenger, 1899 (Ostariophysi: Characiformes: Alestidae). *Folia biologica (Kraków)*. 57:13-21.

Pecio, A. 2009. Ultrastructural examination of spermiogenesis and spermatozoon ultrastructure in Congo tetra Phenacogrammus interruptus Boulenger, 1899 (Ostariophysi: Characiformes: Alestidae). *Folia Biol (Krakow)*. 57:13-21.

Pecio, A., J.R. Burns, and S.H. Weitzman. 2005a. Sperm and spermatozeugma ultrastructure in the inseminating species Tyttocharax cochui, T. tambopatensis, and Scopaeocharax rhinodus (Pisces: Teleostei: Characidae: Glandulocaudinae: Xenurobryconini). *Journal of Morphology*. 263:216-226.

Pecio, A., J.R. Burns, and S.H. Weitzman. 2005b. Sperm and spermatozeugma ultrastructure in the inseminating species Tyttocharax cochui, T. tambopatensis, and Scopaeocharax rhinodus (Pisces: Teleostei: Characidae: Glandulocaudinae: Xenurobryconini). *J Morphol*. 263:216-226.

Pecio, A., J.R. Burns, and S.H. Weitzman. 2007. Comparison of spermiogenesis in the externally fertilizing Hemigrammus erythrozonus and the inseminating Corynopoma riisei (Teleostei: Characiformes: Characidae). *Neotropical Ichthyology*. 5:457-470.

Peňáz, M. 1996. Chondrostoma nasus-its reproduction strategy and possible reasons for a widely observed population decline-a review. *Conservation of endangered freshwater fish in Europe*:279-285.

Pereira, C., L. Barcellos, L.C. Kreutz, R.M. Quevedo, F. Ritter, and L. Silva. 2006. Embryonic and larval development of jundiá (Rhamdia quelen, Quoy & Gaimard, 1824, Pisces, Teleostei), a South American catfish. *Brazilian Journal of Biology*. 66:1057-1063.

Piah, R.M., and D.J. Bucher. 2014. Reproductive Biology of Estuarine Pufferfish, Marilyna pleurosticta and Tetractenos hamiltoni (Teleostei: Tetraodontidae) in Northern NSW: Implications for Biomonitoring. *In* Proceedings of the Linnean Society of New South Wales. Vol. 136.

Pietsch, T.W. 2005. Dimorphism, parasitism, and sex revisited: modes of reproduction among deep-sea ceratioid anglerfishes (Teleostei: Lophiiformes). *Ichthyological Research*. 52:207-236.

Powell, M.L., S.I. Kavanaugh, and S.A. Sower. 2005. Current Knowledge of Hagfish Reproduction: Implications for Fisheries Management1. *Integrative and Comparative Biology*. 45:158-165.

Psenicka, M., S.M. Alavi, M. Rodina, D. Gela, J. Nebesarova, and O. Linhart. 2007. Morphology and ultrastructure of Siberian sturgeon (Acipenser baerii) spermatozoa using scanning and transmission electron microscopy. *Biol Cell*. 99:103-115.

Psenicka, M., S. Hadi Alavi, M. Rodina, Z. Cicova, D. Gela, J. Cosson, J. Nebesarova, and O. Linhart. 2008. Morphology, chemical contents and physiology of chondrostean fish sperm: a comparative study between Siberian sturgeon (Acipenser baerii) and sterlet (Acipenser ruthenus). *Journal of Applied Ichthyology*. 24:371-377.

Pšenička, M., M. Rodina, M. Flajšhans, V. Kašpar, and O. Linhart. 2008. Structural abnormalities of common carp Cyprinus carpio spermatozoa. *Fish Physiology and Biochemistry*. 35:591.

Psenicka, M., M. Rodina, J. Nebesarova, and O. Linhart. 2006. Ultrastructure of spermatozoa of tench Tinca tinca observed by means of scanning and transmission electron microscopy. *Theriogenology*. 66:1355-1363.

Psenicka, M., M. Vancova, P. Koubek, J. Tesitel, and O. Linhart. 2009. Fine structure and morphology of sterlet (Acipenser ruthenus L. 1758) spermatozoa and acrosin localization. *Anim Reprod Sci*. 111:3-16.

Pucci, M.B., P. Barbosa, V. Nogaroto, M.C. Almeida, R.F. Artoni, J.C. Pansonato-Alves, F. Foresti, O. Moreira-Filho, and M.R. Vicari. 2014. Population differentiation and speciation in the genus Characidium (Characiformes: Crenuchidae): effects of reproductive and chromosomal barriers. *Biological Journal of the Linnean Society*. 111:541-553.

PUSEY, B.J., and T. STEWART. 2008. Internal fertilization in Lepidogalaxias salamandroides Mees (Pisces: Lepidogalaxiidae). *Zoological Journal of the Linnean Society*. 97:69-79.

Quagio-Grassiotto, I., F. Antoneli, and C. Oliveira. 2003. Spermiogenesis and sperm ultrastructure in Cichla intermedia with some considerations about Labroidei spermatozoa (Teleostei, Perciformes, Cichlidae). *Tissue and Cell*. 35:441-446.

Quagio-Grassiotto, I., and C. Oliveira. 2008. Sperm ultrastructure and a new type of spermiogenesis in two species of Pimelodidae, with a comparative review of sperm ultrastructure in Siluriformes (Teleostei: Ostariophysi). *Zoologischer Anzeiger-A Journal of Comparative Zoology*. 247:55-66.

Quagio-Grassiotto, I., R. Ortiz, M.S. Pérez, and C. Oliveira. 2011a. Sperm of Doradidae (Teleostei: Siluriformes). *Tissue and Cell*. 43:8-23.

Quagio-Grassiotto, I., R.J. Ortiz, M.H.S. Pérez, and C. Oliveira. 2011b. Sperm of Doradidae (Teleostei: Siluriformes). *Tissue and Cell*. 43:8-23.

Quagio-Grassiotto, I., C. Oliveria, and A. Gosztonyi. 2001. The ultrastructure of spermiogenesis and spermatozoa in Diplomystes mesembrinus. *Journal of fish Biology*. 58:1623-1632.

Quinitio, G.F., and H. Takahashi. 1992. An ultrastructural study on the occurrence of aberrant spermatids in the testis of the river sculpin, Cottus hangiongensis. *Japanese Journal of Ichthyology*. 39:235-241.

Reid, H., and D. Holdway. 1995. Early development of the Australian crimson-spotted rainbowfish, Melanotaenia fluviatilis (Pisces: Melanotaeniidae). *Marine and Freshwater Research*. 46:475-480.

Reist, J.D. 2019. Family Chimaeridae – Shortnose Chimaeras, Chimères. *In* Marine Fishes of Arctic Canada. W.C. Brian and D.R. James, editors. University of Toronto Press. 171-173.

Ribes, E., M. Cheema, R. Gonzalez-Romero, D. Lloris, J. Ausio, and N. Saperas. 2015. Spermiogenesis and biflagellate spermatozoon of the teleost fish Lampanyctus crocodilus (Myctophiformes, Myctophidae): ultrastructure and characterisation of its sperm basic nuclear proteins. *Cell Tissue Res*. 361:619-632.

Riofrio-Quijandría, J.C., S. Flores-Gómez, J. Wasiw, and V. Yépez. 2017. Reproducción, crecimiento y tasa de explotación de Chiochio (Psectrogaster rutiloides) en la cuenca del Río Ucayali entre 2008-2012. *Revista de Investigaciones Veterinarias del Perú*. 28:345-358.
Romagosa, E., P. De Paiva, and H.M. Godinho. 1990. Pattern of oocyte diameter frequency distribution in females of the pacu, Piaractus mesopotamicus (Holmberg 1887)(= Colossoma mitrei Berg 1895), induced to spawn. *Aquaculture*. 86:105-110.
Ruban, G.I., M.V. Kholodova, V.A. Kalmykov, and P.A. Sorokin. 2011. A review of the taxonomic status of the Persian sturgeon (Acipenser persicus Borodin). *Journal of Applied Ichthyology*. 27:470-476.
Rupik, W., J. Huszno, and J. Klag. 2011. Cellular organisation of the mature testes and stages of spermiogenesis in Danio rerio (Cyprinidae; Teleostei)—Structural and ultrastructural studies. *Micron*. 42:833-839.
Rutaisire, J. 2003. The Reproductive Biology and Artificial Breeding of Ningu Labeo Victorianus (Pisces: Cyprinidae.
Sakamoto, T., R.G. Danzmann, N. Okamoto, M.M. Ferguson, and P.E. Ihssen. 1999. Linkage analysis of quantitative trait loci associated with spawning time in rainbow trout (Oncorhynchus mykiss). *Aquaculture*. 173:33-43.
Santana, J.C.d.O., A.L. Netto-Ferreira, D. Calcagnotto, and I. Quagio-Grassiotto. 2013. Sperm characteristics as additional evidence of close relationship between Lebiasina and Piabucina (Characiformes: Lebiasinidae: Lebiasininae). *Neotropical Ichthyology*. 11:573-579.
Santos, H.B.d., E.V. Sampaio, F.P. Arantes, and Y. Sato. 2013. Induced spawning and reproductive variables of the catfish Lophiosilurus alexandri Steindachner, 1876 (Siluriformes: Pseudopimelodidae). *Neotropical Ichthyology*. 11:607-614.
Santos, L.V., C.F.F. Craveiro, A. Soares, L.N. Eduardo, R. Passarone, E.F.B. Silva, and F. Lucena-Frédou. 2021. Reproductive biology of the shorthead drum Larimus breviceps (Acanthuriformes: Sciaenidae) in northeastern Brazil. *Regional Studies in Marine Science*. 48:102052.
Schulz, M., J. Freyhof, R. Saint-Laurent, K. Østbye, T. Mehner, and L. Bernatchez. 2006. Evidence for independent origin of two spring-spawning ciscoes (Salmoniformes: Coregonidae) in Germany. *Journal of Fish Biology*. 68:119-135.
Semple, G. 1991. Reproductive behaviour and early development of the honey blue-eye, <I>Pseudomugil mellis</I> Allen and Ivantsoff 1982 (Pisces : Pseudomugilidae), from the North-east Coast Divison, south-eastern Queensland, Australia. *Marine and Freshwater Research*. 42:277-286.
Shahin, A.A. 2006. Spermatogenesis and spermatozoon ultrastructure in the Nile pebblyfish Alestes dentex (Teleostei: Characiformes: Alestidae) in Egypt. *World Journal of Zoology*. 1:1-16.
Shalaby, F., and H. Migeed. 2012. Impact of environmental contaminants on the testes of Oreochromis niloticus with special reference to ultrastructure of spermatozoa in Lake Manzala (Egypt). *J. Environ. Anal. Toxicol*. 2:1-8.
Shinozaki-Mendes, R.A., F.H.V. Hazin, P.G. De Oliveira, and F.C. De Carvalho. 2007. Reproductive biology of the squirrelfish, Holocentrus adscensionis (Osbeck, 1765), caught off the coast of Pernambuco, Brazil. *Scientia Marina*. 71:715-722.
Smith, W.S., R.C. Biagioni, and L. Halcsik. 2013. Fish fauna of Floresta Nacional de Ipanema, São Paulo State, Brazil. *Biota Neotropica*. 13:175-181.
Sneed, K.E., and H.P. Clemens. 1963. The morphology of the testes and accessory reproductive glands of the catfishes (Ictaluridae). *Copeia*:606-611.
Snickars, M., G. Sundblad, A. Sandström, L. Ljunggren, U. Bergström, G. Johansson, and J. Mattila. 2010. Habitat selectivity of substrate-spawning fish: modelling requirements for the Eurasian perch Perca fluviatilis. *Marine Ecology Progress Series*. 398:235-243.
Song, Y.B., S.R. Oh, J.P. Seo, B.G. Ji, B.S. Lim, Y.D. Lee, and H.B. Kim. 2005. Larval Development and Rearing of Longtooth Grouper Epinephelus bruneus in Jeju Island, Korea. *Journal of the World Aquaculture Society*. 36:209-216.
Spadella, M.A., C. Oliveira, and I. Quagio-Grassiotto. 2012. Spermiogenesis and sperm ultrastructure in ten species of Loricariidae (Siluriformes, Teleostei). *Zoomorphology*. 131:249-263.
Spies, R.B., and D.W. Rice. 1988. Effects of organic contaminants on reproduction of the starry flounder Platichthys stellatus in San Francisco Bay. *Marine Biology*. 98:191-200.
Stanley, H.P. 1971. Fine structure of spermiogenesis in the elasmobranch fish Squalus suckleyi. II. Late stages of differentiation and structure of the mature spermatozoon. *Journal of ultrastructure research*. 36:103-118.

Stanley, H.P. 1983. The fine structure of spermatozoa of Hydrolagus colliei (Chondrichthyes, Holocephali). *Journal of ultrastructure research*. 83:184-194.

Subamia, I.W., and S. Sugito. 2008. EMBRYONIC DEVELOPMENTAL STAGES OF IKAN PALMAS (Polipterus senegalus senegalus). *Indonesian Aquaculture Journal*. 3:119-124.

Tavares-Dias, M., M.S.B. Oliveira, R.A. Goncalves, and L.R. Neves. 2017. Parasitic diversity of a wild Satanoperca jurupari population, an ornamental cichlid in the Brazilian Amazon. *Acta Amazonica*. 47:155-162.

Taylor, M.H., G.J. Leach, L. DiMichele, W.M. Levitan, and W.F. Jacob. 1979. Lunar Spawning Cycle in the Mummichog, Fundulus heteroclitus (Pisces: Cyprinodontidae). *Copeia*. 1979:291-297.

Thompson, M.J. 1974. BURROWING AND BURROW-ASSOCIATED BEHAVIOR IN THE DUSKY JAWFISH, OPISTOGNATHUS WHITEHURSTI. Florida Atlantic University.

Trippel, E.A. 2003. Estimation of male reproductive success of marine fishes. *Journal of Northwest Atlantic Fishery Science*. 33.

Turner, C.L. 1938. Adaptations for viviparity in embryos and ovary of Anableps Anableps. *Journal of Morphology*. 62:323-349.

Tzioumis, V., and M. Kingsford. 1995. Periodicity of spawning of two temperate damselfishes: Parma microlepis and Chromis dispilus. *Bulletin of marine Science*. 57:596-609.

Uehara, S., and M. Shimizu. 1996. Age and growth of stone flounder Kareius bicoloratus in Tokyo Bay, Japan. *Fisheries science*. 62:897-901.

Ueno, K., and B. Arimoto. 1982. Induction of triploids inRhodeus ocellatus ocellatus by cold shock treatment of fertilized eggs. *Experientia*. 38:544-546.

Urbano-Bonilla, A., J. Zamudio, and J.A. Maldonado-Ocampo. 2016. Ecological aspects of Lebiasina erythrinoides (Characiformes: Lebiasinidae) From an Andean piedmont stream in Colombia. *Universitas Scientiarum*. 21:98-114.

Van der Horst, G., and T. Erasmus. 1981. Spawning time and spawning groungs of mullet with special reference to Liza dumerili (Steindachner, 1869). *South African Journal of Science*. 77:73-78.

van der Straten, K.M., B.B. Collette, L.K.-P. Leung, and S.D. Johnston. 2006. Sperm morphology of the black marlin (Makaira indica) differs from scombroid sperm. *Bulletin of Marine Science*. 79:839-845.

Vila, I., and E. Habit. 2015. Current situation of the fish fauna in the Mediterranean region of.

Vischer, H.A. 1989. The development of lateral-line receptors in Eigenmannia (Teleostei, Gymnotiformes). I. The mechanoreceptive lateral-line system (Part 1 of 2). *Brain, behavior and evolution*. 33:205-213.

Wanshu, H., C. Shixi, Z. Qiyong, and W. Qiong. 2007. Reproductive ecology of the mudskipper Boleophthalmus pectinirostris. *Acta Oceanologica Sinica*:72-81.

Watanabe, Y., H. Zenitani, and R. Kimura. 1996. Offshore expansion of spawning of the Japanese sardine, Sardinops melanostictus, and its implication for egg and larval survival. *Canadian Journal of Fisheries and Aquatic Sciences*. 53:55-61.

Wei, Q., P. Li, M. Psenicka, S.H. Alavi, L. Shen, J. Liu, J. Peknicova, and O. Linhart. 2007. Ultrastructure and morphology of spermatozoa in Chinese sturgeon (Acipenser sinensis Gray 1835) using scanning and transmission electron microscopy. *Theriogenology*. 67:1269-1278.

Weitzman, S.H., N.A. Menezes, H.-G. Evers, and J.R. Burns. 2005. Putative relationships among inseminating and externally fertilizing characids, with a description of a new genus and species of Brazilian inseminating fish bearing an anal-fin gland in males (Characiformes: Characidae). *Neotropical Ichthyology*. 3:329-360.

Willis, S.C., M.S. Nunes, C.G. Montaña, I.P. Farias, and N.R. Lovejoy. 2007. Systematics, biogeography, and evolution of the Neotropical peacock basses Cichla (Perciformes: Cichlidae). *Molecular Phylogenetics and Evolution*. 44:291-307.

Xu, G.-H., and L.-J. Zhao. 2016. A Middle Triassic stem-neopterygian fish from China shows remarkable secondary sexual characteristics. *Science Bulletin*. 61:338-344.

Yamaha, E., and F. Yamazaki. 2002. Electrically fused-egg induction and its development in the goldfish, Carassius auratus. *International Journal of Developmental Biology*. 37:291-298.

Yasuda, T., H. Katsumata, R. Kawabe, N. Nakatsuka, and Y. Kurita. 2013. Identifying spawning events in the Japanese flounder Paralichthys olivaceus from depth time-series data. *Journal of Sea Research*. 75:33-40.

Yoon, H.-S., D.-C. Seo, and S.-D. Choi. 2006. Effect of salinity on hatching and spawning characteristics of Miichthys miiuy in the Western of Korea. *Korean Journal of Environmental Biology*. 24:53-59.

Yoon, J.-D., J.-H. Kim, H.-B. Jo, M.-A. Yeom, W.-M. Heo, G.-J. Joo, and M.-H. Jang. 2015. Seasonal habitat utilization and movement patterns of the threatened Brachymystax lenok tsinlingensis in a Korean river. *Environmental Biology of fishes*. 98:225-236.

Zarnescu, O. 2005. Ultrastructural study of spermatozoa of the paddlefish, Polyodon spathula. *Zygote*. 13:241-247.

Zeller, D.C. 1998. Spawning aggregations: patterns of movement of the coral trout Plectropomus leopardus (Serranidae) as determined by ultrasonic telemetry. *Marine Ecology Progress Series*. 162:253-263.

Zhang, L., Y.J. Wang, M.H. Hu, Q.X. Fan, S.G. Chenung, P.K.S. Shin, H. Li, and L. Cao. 2009. Effects of the timing of initial feeding on growth and survival of spotted mandarin fish Siniperca scherzeri larvae. *Journal of Fish Biology*. 75:1158-1172.

Zhang, L., P. Yang, Y. Liu, X. Bian, S. Ullah, Q. Zhang, W. Chen, Y. Le, B. Chen, J. Lin, C. Gao, J. Hu, and Q. Chen. 2014. Pre-spermiogenic initiation of flagellar growth and correlative ultrastructural observations on nuage, nuclear and mitochondrial developmental morphology in the zebrafish Danio rerio. *Micron*. 66:1-8.

ZHANG, Y.-m., and J.-h. JIANG. 2010. A Study on the Artificial Propagation of Acrossocheilus fasciatus [J]. *Journal of Zhejiang Ocean University (Natural Science)*. 3.

Zhao, S., F. Cheng, G. Hou, Z. Hu, and S. Xie. 2019. Opportunistic-tended life history traits of Siniperca kneri in the Three Gorges Reservoir, China: potential responses to impoundment. *Journal of Oceanology and Limnology*. 37:694-705.

Zhu, Z., X. Zeng, X. Lin, Z. Xu, and J. Sun. 2015. Effects of Ration Levels on Growth and Reproduction from Larvae to First-Time Spawning in the Female Gambusia affinis. *International Journal of Molecular Sciences*. 16.
